# Distinguishing *PEX2* and *PEX16* gene variant severity for mild, severe and atypical peroxisome biogenesis disorders

**DOI:** 10.1242/dmm.052258

**Published:** 2025-07-28

**Authors:** Vanessa A. Gomez, Oguz Kanca, Sharayu V. Jangam, Saurabh Srivastav, Jonathan C. Andrews, Michael F. Wangler

**Affiliations:** ^1^ Department of Molecular and Human Genetics, Baylor College of Medicine, Houston, TX 77030, USA; ^2^ Jan and Dan Duncan Neurological Research Institute, Texas Children's Hospital, Houston, TX 77030, USA

**Keywords:** Peroxisome, *Drosophila*, PEX2, PEX16, Zellweger, PBD

## Abstract

Peroxisomal biogenesis disorders (PBD) are autosomal recessive diseases caused by mutations in specific *PEX* genes that impair peroxisome formation, leading to multi-systemic failure. Symptoms vary, even in patients with variants in the same *PEX* gene. Our goal is to select *PEX* mutations and use *Drosophila* to model a severity spectrum based on genotype−phenotype correlations. Utilizing *KozakGAL4 (KZ)* cassettes, we replaced the coding sequence of *Pex* with a *GAL4* driver, ideal for making ‘humanized’ flies in which human *PEX* can replace the fly loss. We generated *Pex2^KZ^* and *Pex16^KZ^* lines and assessed them in various behavior assays, confirming their severe phenotypes. We performed rescue with human reference, variant *PEX2* and *PEX16* alleles, and phenotypic rescue was observed when human *PEX2^Ref^* or *PEX16^Ref^* were expressed in *Pex2^KZ^* or *Pex16^KZ^* flies, respectively. We identified a severity spectrum for *PEX2* and *PEX16* alleles, with some missense mutations exhibiting severity comparable to truncations. Alleles linked to mild PBD showed partial rescue, while variants associated with atypical ataxia could fully rescue. *Drosophila* humanization is an effective method to study the range of severity of PBD.

## Teaser

**Summary:** Analysis of a *Drosophila* model to study rare disease-causing mutations in human *PEX* genes, examining the rescue of peroxisome loss and explaining the severity of disease mutation based on phenotypic outcomes.

## INTRODUCTION

Peroxisomes are ubiquitous organelles that play an important role in cellular metabolism and perform specific biochemical functions in the cell, primarily involving complex lipids in eukaryotic cells (Wanders, 2004; Delille et al., 2006). Important biochemical functions performed by peroxisomes include fatty acid β-oxidation of very-long-chain fatty acids (VLCFAs) (Moser et al., 1984), α-oxidation of branched-chain fatty acids (Poulos et al., 1988; Van Veldhoven, 2010), plasmalogen biosynthesis (Heymans et al., 1983; Braverman and Moser, 2012), and catabolism of reactive oxygen species (Fransen et al., 2012) and glyoxylate (Vandor and Tolbert, 1970; Wanders and Waterham, 2006).

There are two main disorders associated with peroxisomes in humans: peroxisomal biogenesis disorders (PBD) and single enzyme/protein deficiency disorders (SEPD) [see * *Global Foundation for Peroxisomal Disorders (GFPD), https://thegfpd.org/peroxisomal-disorders/]. Peroxisomal biogenesis disorders are a group of autosomal recessive disorders caused by loss-of-function mutations in one of the peroxisomal matrix genes responsible for peroxisomal assembly and function (Wanders, 2004; Wanders and Waterham, 2005). Patients with PBD Zellweger spectrum disorders (ZSD) experience a wide range of multisystemic symptoms often within the first year of life, involving brain, bone, kidney and liver, which can lead to death (Gould and Valle, 2000; Baumgartner and Saudubray, 2002). PBD consists of a spectrum of disorders with varying severity, ranging from mild to moderate to severe phenotypes. Historically, severe PBD-ZSD (also known as Zellweger syndrome) is the most dramatic and rapidly progressive disorder with a high mortality rate (Klouwer et al., 2015; Bose et al., 2022). Intermediate PBD-ZSD (also known as neonatal adrenoleukodystrophy) has an infantile presentation with feeding problems and brain white matter changes, while mild PBD-ZSD (also known as infantile Refsum disease) is marked by hearing loss and retinal degeneration with a much milder cognitive impact (Klouwer et al., 2015; Bose et al., 2022).

The peroxisomal biogenesis machinery is a conserved process, which is highly dependent on the action of 14 peroxins encoded by *PEX* genes that are required for matrix protein import, peroxisome membrane assembly and peroxisome proliferation (Subramani, 1998; Fujiki et al., 2014). Early peroxisomal proteins, including *PEX3*, *PEX19* and *PEX16*, aid in the designation of an ER-derived lipid bilayer and its maturation to a pre-peroxisomal vesicle (Subramani, 1998). Membrane proteins are then incorporated to allow enzyme import into the maturing peroxisome with the help of *PEX2* and the importer complex (Platta et al., 2009). Mature peroxisomes can then perform a wide range of biochemical functions important for cell function and proper metabolism (Wanders, 2004; Delille et al., 2006). Therefore, it is critical to study *PEX* genes involved in different aspects of biogenesis when characterizing peroxisomal biogenesis disorders, such as *PEX2* that is important for matrix protein import, and *PEX16* that is important for early peroxisomal formation.

The severity spectrum of PBD-ZSD correlates to the allele severity of the *PEX* gene mutations. In general, clinical severity correlates to lower levels of residual *PEX* gene activity and much less to which *PEX* gene is involved (e.g. *PEX16* vs *PEX2* vs *PEX3*) (Rosewich et al., 2005; Waterham and Ebberink, 2012). Patients with biallelic loss of function ‘null’ *PEX* alleles are more likely to exhibit drastic biochemical defects and severe cases of PBD-ZSD than patients harboring one of the hypomorphic alleles that retain partial PEX protein functions. For instance, milder variant alleles of *PEX2*, such as *PEX2^E55K^*, in compound heterozygotes *(PEX2^Null^/PEX2^E55K^)* are associated with mild PBD-ZSD, with phenotypes being mild or intermediate due to residual *PEX2* function (Imamura et al., 1998; Shimozawa et al., 1999). Likewise, we have previously observed a case comprising a single amino acid deletion of *PEX16* with normal plasma VLCFAs and an atypical ataxia phenotype (Bacino et al., 2015a,b). In summary, PBD-ZSD exhibits a clinical spectrum that correlates with specific alleles.

Peroxisomal biology is highly conserved across eukaryotes, and recent studies have demonstrated the evolutionary conservation of peroxisomal biogenesis in *Drosophila* (Chen et al., 2010; Mast et al., 2011; Nakayama et al., 2011; Faust et al., 2012, 2014; Wangler et al., 2017). Previous studies have found that *Pex* mutations in *Drosophila* alter lipid metabolism, muscle function and spermatogenesis (Chen et al., 2010; Faust et al., 2012, 2014). Our research has focused on *Pex2* and *Pex16* mutant flies, which allowed us to compare different biogenesis defects. We have previously documented a detailed phenotypic characterization in flies on two alleles for *Pex2* and *Pex16*, studying the mutants in trans with genomic deficiencies and creating genomic rescue strains for each mutation (Wangler et al., 2017). We also have shown that the peroxisomes are similarly functionally and morphologically defective in *Pex2* or *Pex16* mutants, with mutant flies having short lifespans, increased bang sensitivity, lacking flight ability, and showing reduced activity (Wangler et al., 2017). Additionally, functional analysis of peroxisomal lipids allowed for a comprehensive study of VLCFA metabolites in different stages of development, as well as findings of a dramatic loss of plasmalogen synthesis in both *Pex2* and *Pex16* mutant flies (Wangler et al., 2017). Altogether, previous studies of peroxisomes in *Drosophila* support the high conservation of the peroxisomal biogenesis machinery in flies and humans.

A key question that has not been addressed previously is whether human transgenes can rescue deleterious *Pex* mutant phenotypes in flies. While there has been some functional characterization of peroxisomal biogenesis machinery in flies, studies on known human disease-causing variants involved in PBD are more limited. The human *PEX1* G843D variant has been studied in *Drosophila* for a pharmacologic screen (Liu et al., 2021). Studies of specific *PEX* gene mutations in flies have great potential. Humanization in *Drosophila* allows for a human gene to be expressed in the mutant background in the same spatial and temporal pattern as the fly gene (Bellen and Yamamoto, 2015). Here, we utilize *KozakGAL4* cassettes to knock-out *Pex* genes of interest, creating an effective null background that allowed us to express human reference *PEX2^Ref^;Pex2^KZ^/Pex2^2^* and *PEX16^Ref^;Pex16^KZ^/Pex16^1^*, as well as variant UAS cDNAs of the targeted gene to asses the rescue of these *Pex* mutants, such as *PEX2^E55K^;Pex2^KZ^/Pex2^2^*, PEX2^R119^*;Pex2^KZ^/Pex2^2^, PEX2^W223^*;Pex2^KZ^/Pex2^2^, *PEX2^C247R^;Pex2^KZ^/Pex2^2^*, PEX16^R176^*;Pex16^KZ^/Pex16^1^, and *PEX16^F332del^;Pex16^KZ^/Pex16^1^*. We then assayed the flies for phenotypes allowing us to study disease progression resulting from the expression of the human variants that could be classified as pathogenic or likely pathogenic. We also studied whether the human protein can functionally replace the loss of the endogenous fly Pex protein. With these findings, we identified a method to functionally characterize individual human *PEX* mutations in *Drosophila* and create an allelic series.

## RESULTS

### Generation of the *KozakGAL4* lines resulting in a *GAL4* gene-trap allele and examination of the spatiotemporal expression pattern

To study the neurodevelopmental and neurodegenerative phenotypes seen in PBD patients, we generated novel alleles for our genes of interest with *KozakGAL4* cassettes in *Drosophila.* This strategy replaces the coding sequence of genes with the *KozakGAL4* cassette by using CRISPR-induced homologous recombination, and single guide RNAs (sgRNAs) targeting the 5′ and 3′ UTR (Kanca et al., 2022). These alleles are useful for determining expression pattern of a gene, studying the effect of loss-of-function of the gene product, assessing protein subcellular localization with the help of a GFP protein trap, and expression of UAS-cDNAs of the targeted gene and variants to assess rescue of the mutant phenotypes (Kanca et al., 2022).

**Fig. 1.**
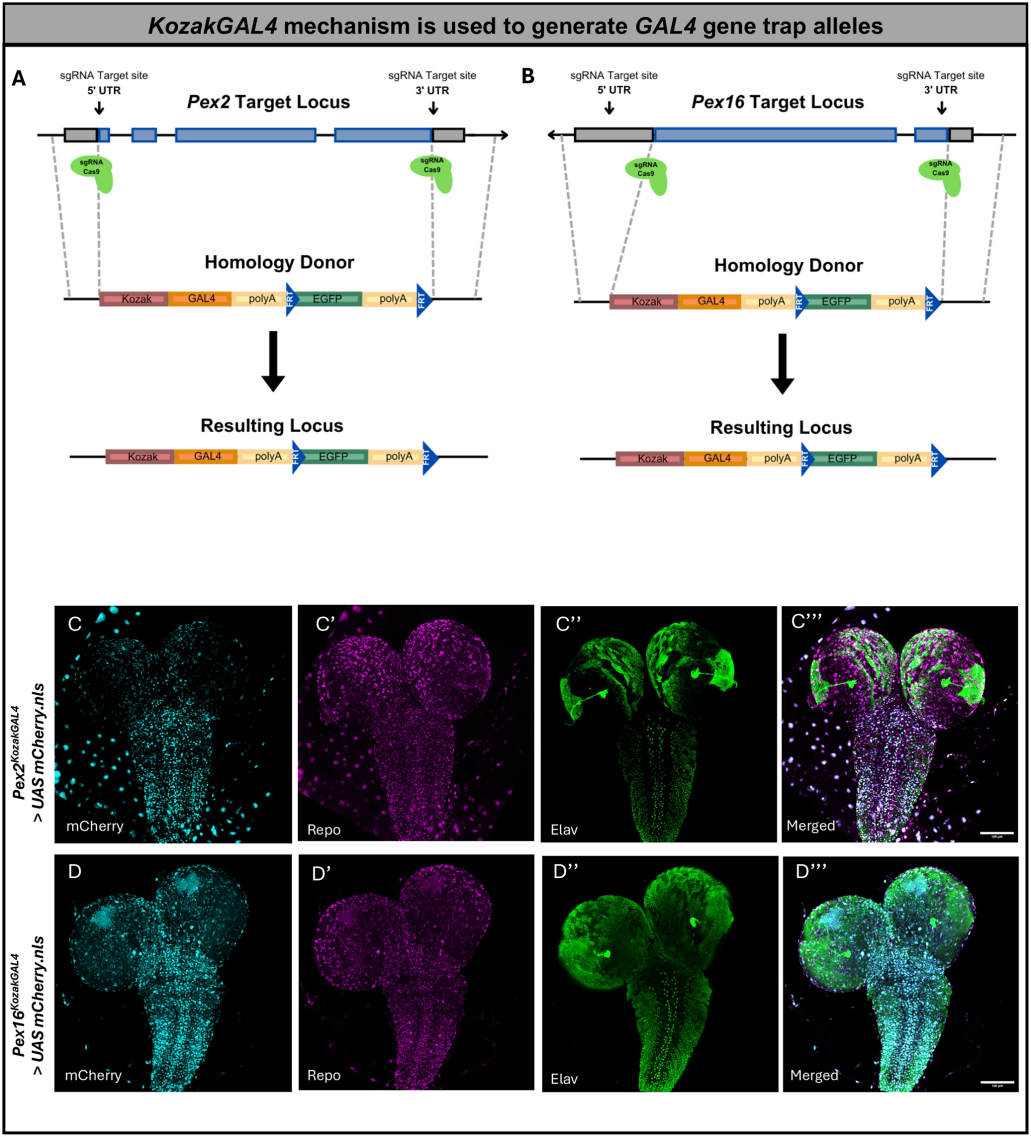
**The *KozakGAL4* knock-in/knock-out strategy, which replaces the coding region of the *Pex* gene of interest by identifying sgRNA target sites within the 5′ UTR and 3′ UTR.** (A) Schematics of *Pex2* locus and targeting strategy. (B) Schematics of *Pex16* locus and targeting strategy. Gray boxes in A and B indicate UTRs; blue boxes indicate the *Pex*-coding region. (C-C″) Immunofluorescence images showing the *Pex2^KZ^* expression pattern in third-instar *Drosophila* larval brains. *Pex2^KZ^* expression (mCherry, blue) is shown in C. Staining against Repo (nuclear glia cells, purple) or Elay (neurons, green) is shown in C′ or C″, respectively. (C‴) Merged image showing colocalization within the ventral nerve cord, indicating *Pex2^KZ^* (blue) expression in both neurons (green) and nuclear glia (purple) of the larval brain. (D-D″) Immunofluorescence images showing the *Pex16^KZ^* expression pattern in third-instar *Drosophila* larval brains. *Pex16^KZ^* expression (mCherry, blue) is shown in D. Staining against Repo (nuclear glia cells, purple) or Elav (neurons, green) is shown in D′ or D″, respectively. (D‴) Merged image showing colocalization within the ventral nerve cord, indicating *Pex16^KZ^* (blue) expression in both neurons (green) and nuclear glia (purple) in the larval brain. Scale bars: 100 µm.

We generated fly lines in which the coding sequence of *Pex2* and *Pex16*, respectively, was replaced by the *KozakGAL4* gene-trap sequence (Fig. 1A,B) and confirmed the insertions by PCR. This technology led to a null allele (*Pex2^KZ^* and *Pex16^KZ^*) with expression of *GAL4* in a similar spatial and temporal pattern as the protein encoded by the targeted gene (Kanca et al., 2022). This method has been successfully employed to study expression patterns and to humanize the gene by driving the human cDNA to effectively replace the gene loss in the fly (Dutta et al., 2024; Yamamoto et al., 2024).

We examined the expression using third-instar larval *Drosophila* brain (Fig. 1C,D) and assessed whether the *Pex2* and *Pex16* genes were expressed in neurons, glia or both, by co-staining for neuronal expression (Elav) and nuclear glia (Repo) proteins. Within the ventral nerve cord (VNC), we observed co-staining of cells expressing *Pex2* or *Pex16* for Repo and Elav (Fig. 1C,D). We noted that *Pex2* expression was generally sparser in the larval third-instar brain. Altogether, *Pex2* and *Pex16* appear to be expressed in both neurons and glia within the larval brain.

### The novel alleles *Pex2^KZ^* and *Pex16^KZ^* behave similar to known *Pex2* and *Pex16* null alleles

**Fig. 2.**
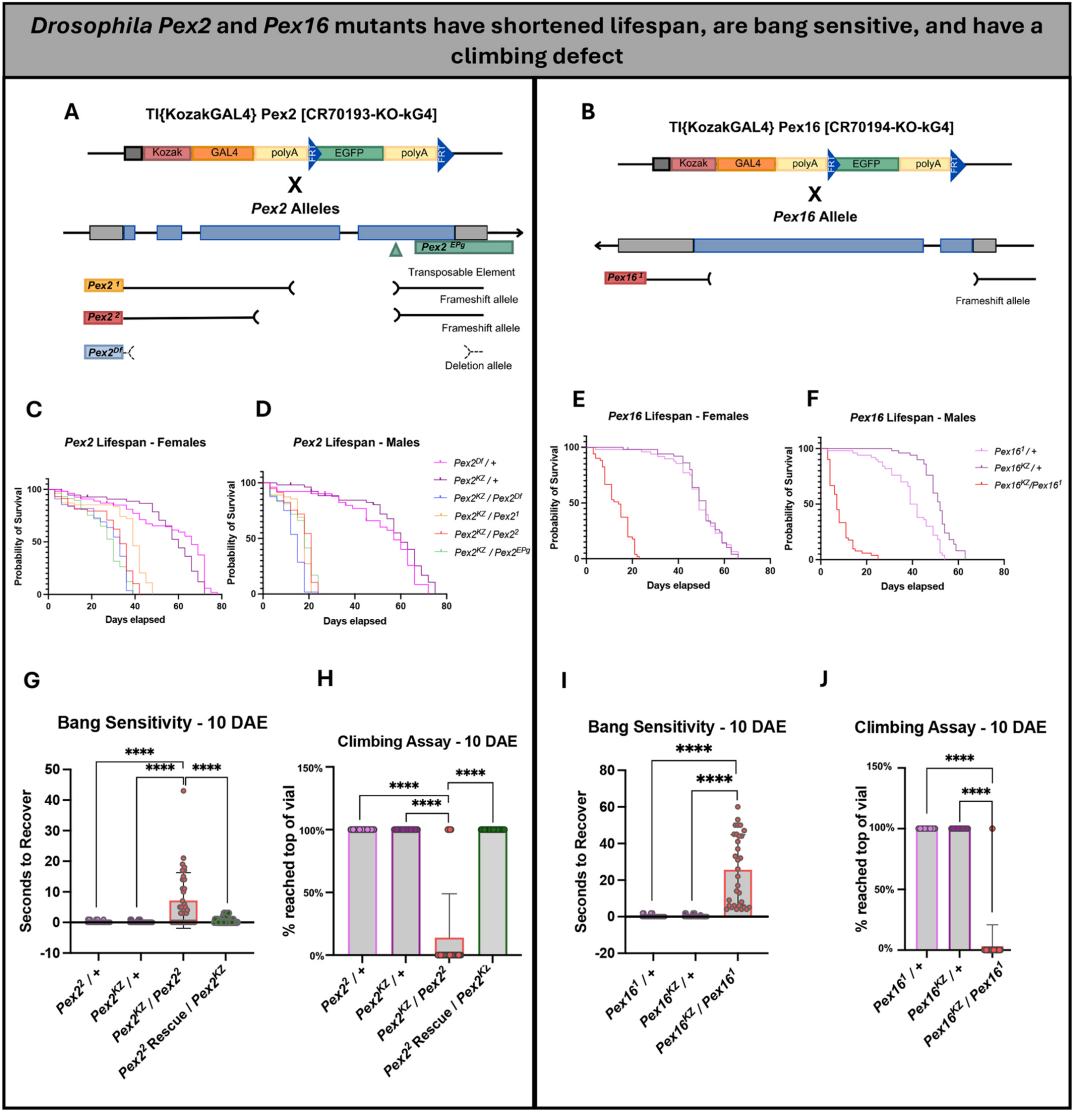
***Drosophila Pex2* and *Pex16* mutants have shortened lifespan, are bang sensitive and have a climbing defect.** (A) Schematic representation of the fly *Pex2* gene along with four alleles, including a coding P-element insertion (*Pex2^EPg^*), two deletion alleles (*Pex2^1^*, *Pex2^2^*) and *Pex2*-*KozakGAL4 (Pex2^KZ^*). (B) Schematic representation of the fly *Pex16* gene along with one frameshift alleles (*Pex16^1^*) and *Pex16*-*KozakGAL4 (Pex16^KZ^*). (C) *Pex2* female lifespan assay shows that the *Pex2* mutants have a shorter lifespan compared to Pex2 control lines (*n*=51 *Pex2*^*Df*^*/*+ and *n*=50 *Pex2*^*KZ*^/+). *n*=55 *Pex2^KZ^/Pex2^Df^*, *n*=54 *Pex2^KZ^/Pex2^1^*, *n*=58 *Pex2^KZ^/Pex2^2^*, *n*=57 *Pex2^KZ^/Pex2^EPg^*. (D) *Pex2* male lifespan assay shows that the *Pex2* mutants have a shorter lifespan compared to Pex2 control lines (*n*=37 *Pex2*^*Df*^*/*+ and *n*=48 *Pex2*^*KZ*^/+). *n*=56 *Pex2^KZ^/Pex2^Df^*, *n*=55 *Pex2^KZ^/Pex2^1^*, *n*=57 *Pex2^KZ^/Pex2^2^*, *n*=53 *Pex2^KZ^/Pex2^EPg^*. (E) *Pex16* female lifespan assay shows that the *Pex16*^*KZ*^*/Pex16*^*1*^ mutant flies (*n*=51) have a shorter lifespan compared to *Pex16* control lines (*n*=48 *Pex16*^*1*^*/*+ and *n*=50 *Pex16*^*KZ*^/+). (F) *Pex16* male lifespan assay shows that the *Pex16*^*KZ*^*/Pex16*^*1*^ mutant flies (*n*=51) have a shorter lifespan compared to *Pex16* control lines (*n*=50 *Pex16*^*1*^*/*+ and *n*=50 *Pex16*^*KZ*^/+). (G) *Pex2* null flies have a significant bang-sensitive phenotype (*Pex2*^*KZ*^*/Pex2*^*2*^, *n*=36) compared to *Pex2* control lines (*n*=40 *Pex2*^*2*^*/*+ and *n*=40 *Pex2*^*KZ*^/+) and *Pex2^2^* rescue (*n*=29) observed at 10 days after eclosion (DAE). (H) *Pex2* null flies have a significant climbing deficiency (*Pex2*^*KZ*^*/Pex2*^*2*^, *n*=36) compared to *Pex2* control lines (*n*=40 *Pex2*^*2*^*/*+ and *n*=40 *Pex2*^*KZ*^/+) and *Pex2^2^* rescue (*n*=29) observed at 10 DAE. (I) *Pex16* null flies have a significant bang-sensitive phenotype (*Pex16*^*KZ*^*/Pex16*^*1*^, red *n*=32) compared to *Pex16* control lines (*n*=40 *Pex16*^*1*^*/*+ and *n*=40 *Pex16*^*KZ*^/+) observed at 10 DAE. (J) *Pex16* null flies have a significant climbing deficiency (*Pex16*^*1*^*/Pex16*^*KZ*^, *n*=32) compared to *Pex16* control lines (*n*=40 *Pex16*^*1*^*/*+ and *n*=40 *Pex16*^*KZ*^/+) observed at 10 DAE. ***P<0.001, ****P<0.0001.

Having generated these unique *KozakGAL4* cassettes, we wanted to study the strength of these loss-of-function alleles of *Pex2* and *Pex16* using behavior assays. We crossed *Pex2^KZ^* to known *Pex2* null alleles, including a transposable element, a deletion allele, and two frameshift alleles *Pex2^1^* and *Pex2^2^* (Fig. 2A). Similarly, we crossed *Pex16^KZ^* to *Pex16^1^*, a known *Pex16* null allele (Fig. 2B).

We first analyzed the lifespan of the F1 progeny of the flies crossed from *Pex2^KZ^* to the four known *Pex2* null alleles. Lifespan analysis showed a dramatic difference between female *Pex2* experimental flies and controls (Fig. 2C). To compare the survival curves between the mutant flies and the *Pex2^KZ^*/+ control, we performed the Log-rank (Mantel−Cox) curve significance test and found a significant difference between the *Pex2* mutant flies and controls (Table S1A). This effect was even more dramatic in males (Fig. 2D). In parallel, we performed lifespan analysis on the F1 progeny of the flies crossed from *Pex16^KZ^* to the *Pex16^1^* null allele (Fig. 2E,F). Lifespan analysis showed a significant difference between the *Pex16^KZ^/Pex16^1^* mutant and controls in both females and males (Table S1B).

We also assessed behavior assays, first focusing on the *Pex2^KZ^/Pex2^2^* mutant flies compared to controls. Bang sensitivity is an assay used to test for seizure-like behavior and paralysis following mechanical stimulation (Fergestad et al., 2006). 10 days after eclosion (DAE), the *Pex2^KZ^/Pex2^2^* mutant flies were found to have a significant bang-sensitive phenotype compared to heterozygous controls (Fig. 2G). Additionally, we generated a P[acman] (https://flypush.research.bcm.edu/pacmanfly/pacman.html) genomic rescue construct (*Pex2^2^* Rescue) to observe rescue of the phenotypes observed in *Drosophila Pex2^KZ^/Pex2^2^* null flies, and we found complete rescue of the bang-sensitive phenotype at 10 DAE (Fig. 2G). Additionally, the climbing assay evaluates the natural tendency of flies to climb, which is known as negative geotaxis (Madabattula et al., 2015). In *Drosophila*, negative geotaxis relies on the presence of intact sensory and motor systems, and a defect in climbing may be an effective readout for peroxisomal disease progression in the fly (Madabattula et al., 2015). When observing the climbing behavior of the *Pex2^KZ^/Pex2^2^* flies compared to heterozygous controls and the *Pex2^2^* Rescue construct at 10 DAE, we observed that only 18% of *Pex2* null flies had the ability to climb a full 8 cm within 60 s, while all control and rescue construct flies were able to climb (Fig. 2H). This finding demonstrates a clear lack of climbing ability and putative disease progression.

We also assessed bang sensitivity and climbing defects on *Pex16* mutant flies compared to controls. When performing the bang-sensitivity assay on *Pex16^KZ^/Pex16^1^* mutant flies, we found a significant seizure-like phenotype that was not seen in the heterozygous control flies (Fig. 2I). When assessing climbing ability on *Pex16* mutant flies 10 DAE, only 3% of the *Pex16^KZ^/Pex16^1^* flies were able to climb the full 8 cm within 60 s (Fig. 2J). In summary, the *KozakGAL4* cassette alleles *Pex2^KZ^* and *Pex16^KZ^* behave as genetic null alleles, similar to previously documented deletions of *Pex2^2^* and *Pex16^1^* in flies (Wangler et al., 2017).

### Rescue-based humanization of *Drosophila Pex* genes to study rare *PEX* variants

**Fig. 3.**
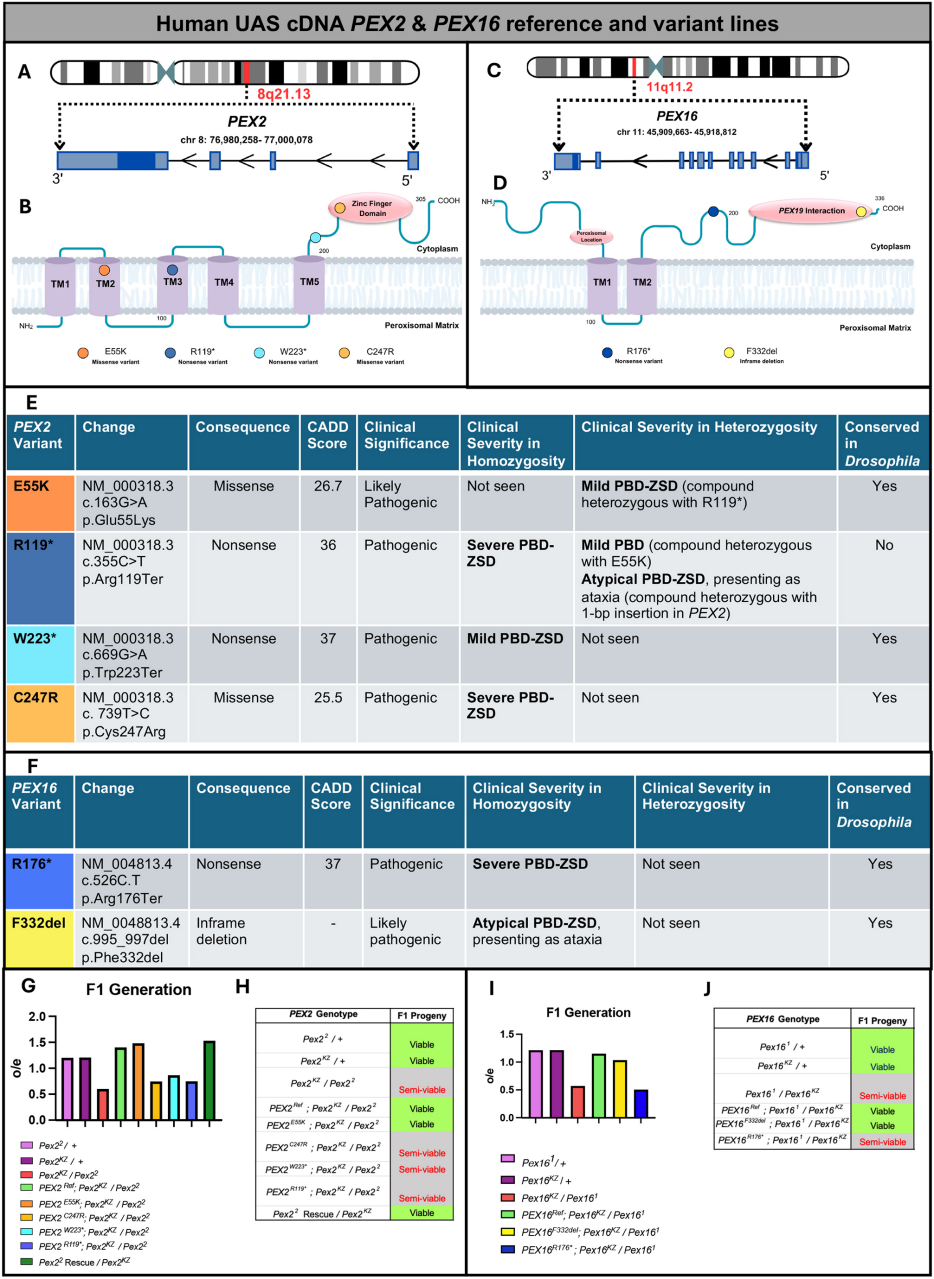
**Human UAS cDNA *PEX2* and *PEX16* reference and variant lines.** (A) Schematic representation of human *PEX2* gene. (B) Schematic representation of human PEX2 protein and variant locations. (C) Schematic representation of human *PEX16* gene. (D) Schematic representation of human PEX16 protein and variant locations. (E) *PEX2* variant table indicates the consequence of the change, pathogenicity prediction, clinical significance, clinical severity in homozygosity and heterozygosity, and conservation in *Drosophila.* (F) *PEX16* variant table indicates the consequence of the change, pathogenicity prediction, clinical significance, clinical severity in homozygosity and heterozygosity, and conservation in *Drosophila.* (G) Plotted are the observed/expected Mendelian ratios of the F1 generation of human *PEX2* variants, in a fly null background. *n*=306 *Pex2*^*2*^*/+*, *n*=345 *Pex2*^*KZ*^*/+*, *n*=273 *Pex2^KZ^/Pex2^2^*, *n*=214 *PEX2^Ref^*, *n*=135 *PEX2^E55K^*, *n*=172 *PEX2^C247R^*, *n*=365 PEX2^W223^*, *n*=337 PEX2^R119*^, *n*=136 *Pex2^2^* Rescue*/Pex2^KZ^.* (H) Assessment of the *PEX2* phenotype and its F1 progenies identified as lethal, viable, or semi-viable. (I) Plotted are the observed/expected Mendelian ratios of the F1 generation of human *PEX16* variants, in a fly null background. *n*=292 *Pex16*^*1*^*/+*, *n*=270 *Pex16*^*KZ*^*/+*, *n*=126 *Pex16^KZ^/Pex16^1^*, 278 *PEX16^Ref^*, 197 P*EX16^F332del^*, 214 PEX16^R176*^. (J) Assessment of the *PEX16* phenotype and its F1 progenies identified as lethal, viable or semi viable. For the calculation of the observed/expected Mendelian ratios of the F1 generations see Table S3.

Having established that the *Pex2^KZ^* and *Pex16^KZ^* are strong loss-of-function alleles and express transgene under GAL4-UAS system, we chose to further study rare human variants involved in PBD and focus further on human *PEX2* and *PEX16*. *PEX2* is found on chromosome 8 (Fig. 3A), and contains five transmembrane domains and a Zinc finger binding domain (Fig. 3B). *PEX16* is found on chromosome 11 (Fig. 3C), and contains two transmembrane domains, a peroxisomal location domain and a *PEX19* interaction domain (Fig. 3D). The rescue-based humanization experiments of *Pex2* and *Pex16* involve the generation of transgenic *Drosophila* lines, containing human reference and variant *PEX2* and *PEX16* under the control of the UAS. ‘Humanization’ was performed by expressing the reference or variant UAS-human cDNA in either the *Pex2^KZ^/Pex2^2^* or *Pex16^KZ^/Pex16^1^* mutant background as appropriate, and by observing for differences in phenotype that suggest a *Pex*-specific effect (Bellen and Yamamoto, 2015; Harnish et al., 2019).

Autosomal recessive mutations in *PEX2* have been associated with a range of mild to intermediate to severe cases of PBD-ZSD. We selected four variants that span the range of clinical severity to test in *Drosophila* (Shimozawa et al., 1992, 1999; Mandel et al., 1994; Imamura et al., 1998; Fujiwara et al., 2000; Gootjes et al., 2004; Mignarri et al., 2012). We generated transgenic *Drosophila* lines with the four selected human variants, as well as a human reference line, designed to express the human protein in *Drosophila* (codon optimized for *Drosophila* genome) with a C-terminal GFP tag (Fig. 3E). Our first variant *PEX2^E55K^* was classified on ClinVar (https://www.ncbi.nlm.nih.gov/clinvar/) (Variation ID:13705) as a likely pathogenic variant in an individual with mild PBD-ZSD who was compound heterozygous with a pathogenic missense variant in PEX2^R119^* (Imamura et al., 1998; Shimozawa et al., 1999). Experimental evidence from cells from patients with this particular *PEX2* variant demonstrates that the *PEX2^E55K^* variant displays residual enzymatic activity and is competent to import target proteins into peroxisomes (Shimozawa et al., 1999; Fujiwara et al., 2000). The *PEX2^E55K^* cells also demonstrate peroxisomal mosaicism in cell culture, where some cells within the culture do not contain peroxisomes (Shimozawa et al., 1999; Fujiwara et al., 2000). This feature is thought to be related to the mild temperature sensitivity of the *PEX* allele and presumed stochastic factors within the cell culture (Shimozawa et al., 1999; Fujiwara et al., 2000). PEX2^R119^* is noted on ClinVar (Variation ID: 13704) as a pathogenic variant in several PBD-ZSD cases and is known to alter peroxisome assembly (Shimozawa et al., 1992). When homozygous, the PEX2^R119^* variant has been shown to cause a severe PBD-ZSD (Zellweger syndrome) phenotype and death in early infancy (Gootjes et al., 2004). When seen in cases with other PBD-ZSD variants, the clinical severity of the PEX2^R119^* variant varies based on the other allele, with findings of mild PBD-ZSD with PEX2^R119^*/ PEX2^E55K^ and findings of mild PBD-ZSD manifesting as childhood-onset cerebellar ataxia and an axonal sensorimotor polyneuropathy with PEX2^R119^* and a 1-bp insertion (c.865_866insA;170993.0006) in the *PEX2* gene (Shimozawa et al., 1999; Mignarri et al., 2012). The PEX2^W223^* variant is also noted on ClinVar (Variation ID: 139590) as a pathogenic variant. The proband, who was homozygous for the PEX2^W223^* variant, displayed mild PBD-ZSD and had normal infancy but, by the age of 22 months, he had hypotonia, cerebellar and vermian atrophy, and continued to deteriorate until he died at age 13 (Mandel et al., 1994; Gootjes et al., 2004). The last *PEX2* variant generated was *PEX2^C247R^* mutation, which is also noted on ClinVar (Variation ID: 139589) as a pathogenic variant. This variant was seen in a newborn patient with severe PBD-ZSD (Zellweger syndrome), with a low birth weight, severe hypotonia, seizures, absent corpus callosum, severe icterus and absent peroxisomes, leading to death at 3 months of age (Gootjes et al., 2004).

We also followed a similar strategy to study human variants for *PEX16*. We have generated two transgenic *Drosophila* lines (codon optimized for *Drosophila genome*) with human variants of varying clinical severity, as well as a *PEX16* human reference line (Fig. 3F). Our variant PEX16^R176^* is interpreted as a pathogenic variant on ClinVar (Variation ID: 6466) and is expected to result in an absent or disrupted protein. This variant has been observed as homozygous or in trans with another pathogenic variant in patients with the hallmark clinical features of Zellweger syndrome (Honsho et al., 1998; Ohba et al., 2013). We also generated a transgene for a unique case that we have previously reported. The patient was homozygous for a *PEX16^F332del^* variant that is currently interpreted as likely pathogenic on ClinVar (Variation ID: 209181) (Bacino et al., 2015a,b; Posey et al., 2016). Our proband had no symptoms until the age of seven and then displayed a progressive ataxia phenotype that was undiagnosed for 18 years, as the patient had multiple plasma studies, including peroxisomal biochemical assays that had normal findings (Bacino et al., 2015a,b).

Once we generated the reference and variant lines, we then crossed these lines to the corresponding *Pex2* or *Pex16* fly null background and generated flies that were compound heterozygous for either *Pex2^KZ^/Pex2^2^* or *Pex16^KZ^/Pex16^1^*, expressing the human transgenes. We had previously observed that *Pex2^KZ^/Pex2^2^* without transgenes were semi-viable, leading to ∼50% of the expected progeny by Mendelian ratios, while controls and the P[acman] genomic rescue construct (*Pex2^2^* Rescue) were over 100% viable (Fig. 3G). The *PEX2^Ref^* human transgene was able to fully rescue this semi-viability. Interestingly, *PEX2^E55K^* also fully rescued, but the *PEX2^C247R^*, PEX2^W223^* and PEX2^R119^* transgenes did not rescue the semi-viability, although PEX2^W223^* was >80% observed/expected (Fig. 3G,H). We also utilized the *PEX16* variants along with the reference line in *PEX16* to document rescue of semi-viability of *Pex16^KZ^/Pex16^1^* and found complete rescue by *PEX16^Ref^* (Fig. 3I). For the two *PEX16* alleles, we saw no rescue of semi-viability for PEX16^R176^*, but *PEX16^F332del^* fully rescued (Fig. 3I,J).

### Human *PEX* reference gene rescues fly peroxisome morphology

**Fig. 4.**
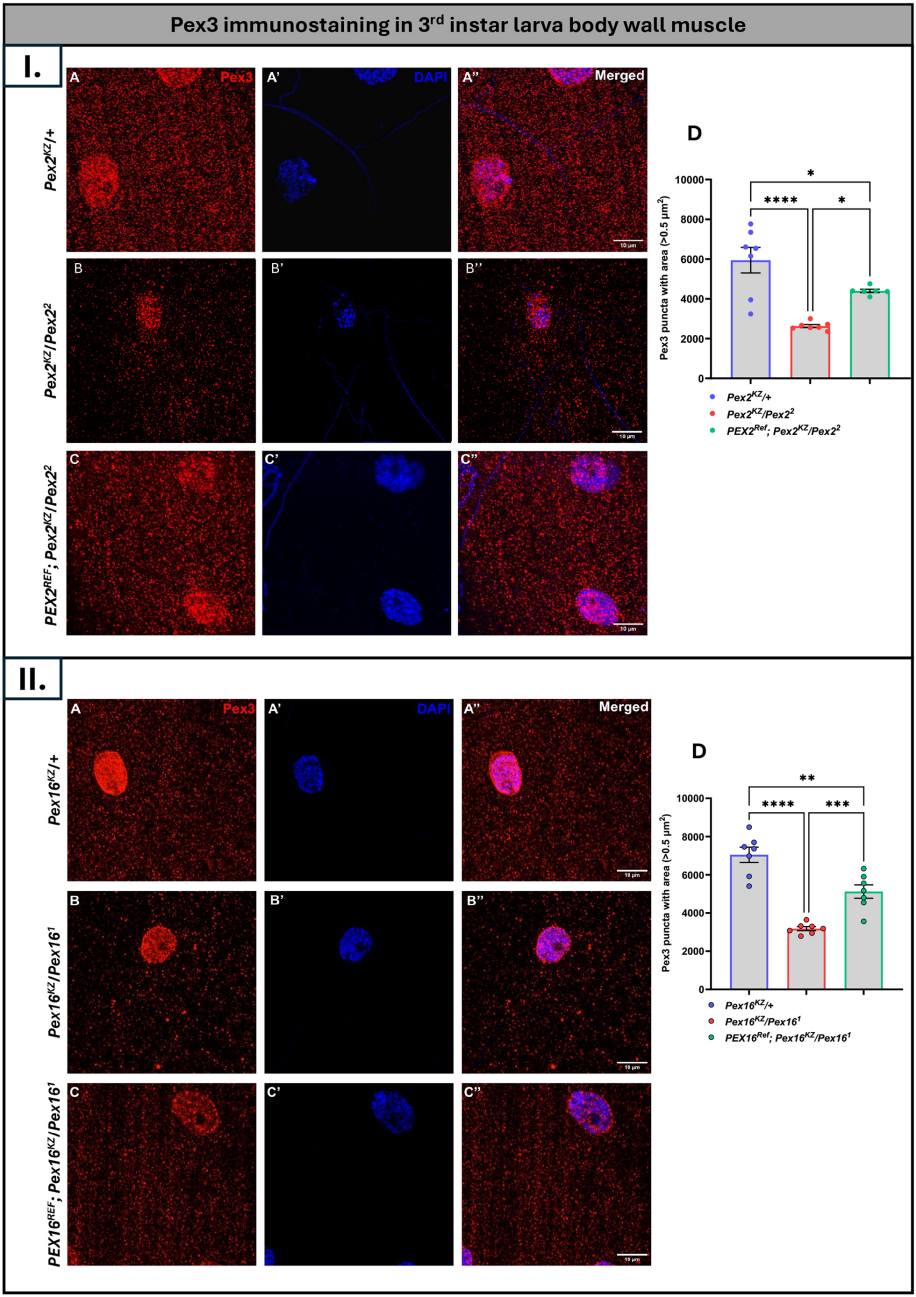
**Pex3 immunostaining in third-instar larva body wall muscle.** (I, top) Images show control group flies (*Pex2^KZ^/+*) (A-A″), *Pex2* null flies (*Pex2^KZ^/Pex2^2^*), (B-B″) and human rescue group flies (*PEX2^Ref^*;*Pex2^KZ^/Pex2^2^*) (C-C″). Pex3-positive puncta are shown in red; nuclei were stained with DAPI (blue). Merged images are shown in A″-B″. (D) Quantification and comparison of flies shown in A-C″ with Pex3-positive puncta comprising an area >0.5µm^2^. *n*=7 *Pex2^KZ^/+* (control), *n*=7 *Pex2^KZ^/Pex2^2^*, *n*=6 *PEX2^Ref^*;*Pex2^KZ^/Pex2^2^*. (II, bottom) Images show control group flies (*Pex16^KZ^/+*) (A-A″), *Pex16* null flies (*Pex16^KZ^/Pex16^2^*) (B-B″) and human rescue group flies (*PEX16^Ref^*;*Pex16^KZ^/Pex16^2^*) (C-C″). Pex3-positive puncta are shown in red; nuclei were stained with DAPI (blue). Merged images are shown in A″-B″. (D) Quantification and comparison of flies shown in A-C″ with Pex3-positive puncta comprising an area >0.5 µm^2^ between the three genotypes. *n*=7 *Pex16^KZ^/+* control, *n*=7 *Pex16^KZ^/Pex16^1^*, *n*=7 *PEX16^Ref^*. *P<0.05, **P=0.0011, ***P=0.001, ****P<0.0001. All scale bars: 10 µm

Next, we wanted to test the impact of the human proteins on rescue to peroxisomal morphology defects (Fig. 4). We utilized anti-Pex3 staining in the third-instar larval body wall muscle. The Pex3 antibody stains the early pre-peroxisomal vesicles, and we have previously observed that, in *Pex2* null and *Pex16* null mutants, Pex3 puncta are present but their number is significantly reduced with a more severe reduction in *Pex16* mutants (Faust et al., 2014; Wangler et al., 2017).

In the *Pex2* control line, we saw Pex3 staining in a punctum pattern throughout the body wall (Fig. 4I, panel A). For *Pex2^KZ^/Pex2^2^* mutants, we observed a dramatic reduction in the number of Pex3 puncta compared to control (*Pex2^KZ^/+*) (Fig. 4I, panel B). This peroxisomal morphology phenotype was partially rescued by the human *PEX2* expression (*PEX2^Ref^*;*Pex2^KZ^/Pex2^2^* flies) (Fig. 4I, panel C). The Pex3 reduction in the *Pex2^KZ^/Pex2^2^* mutant is dramatic and statistically significant (*P*<0.0001) providing further evidence that the *Pex2^KZ^* is a strong loss-of-function allele (Fig. 4I, panel D). Expression of the human reference *PEX2* transgene expression leads to a robust increase in the number of Pex3 puncta in *PEX2^Ref^*;*Pex2^KZ^/Pex2^2^* flies compared to *Pex2^KZ^/Pex2^2^* mutants, but punctum numbers are also significantly less than for the control, indicating a partial rescue (Fig. 4I, panel D). In the *Pex16* lines, we also saw a dramatic loss in the number of Pex3 puncta in the mutant compared to the control, and this phenotype was also partially rescued by the human *PEX16* transgene (*PEX16^Ref^*;*Pex16^KZ^/Pex16^2^* flies) (Fig. 4II, panels A-D). Of note, some background staining was observed when using the Pex3 antibody, which is included in several of our papers on fly peroxisomes (Wangler et al., 2017). Our current understanding is that this is an artifact and cross reactivity, as Pex3 is not known to localize to the nucleus.

In summary, both human *PEX2* and *PEX16* human genes can function in *Drosophila* and partially rescue Pex3 staining, indicating that the human genes can successfully restore peroxisomes in a fly mutant. Therefore, we have generated humanized flies for *PEX2* and *PEX16* in which disease-causing variants and other phenotypes can be tested.

### Human *PEX* reference partially rescues fly *Pex* null behavior phenotypes but the variants fail to do so

**Fig. 5.**
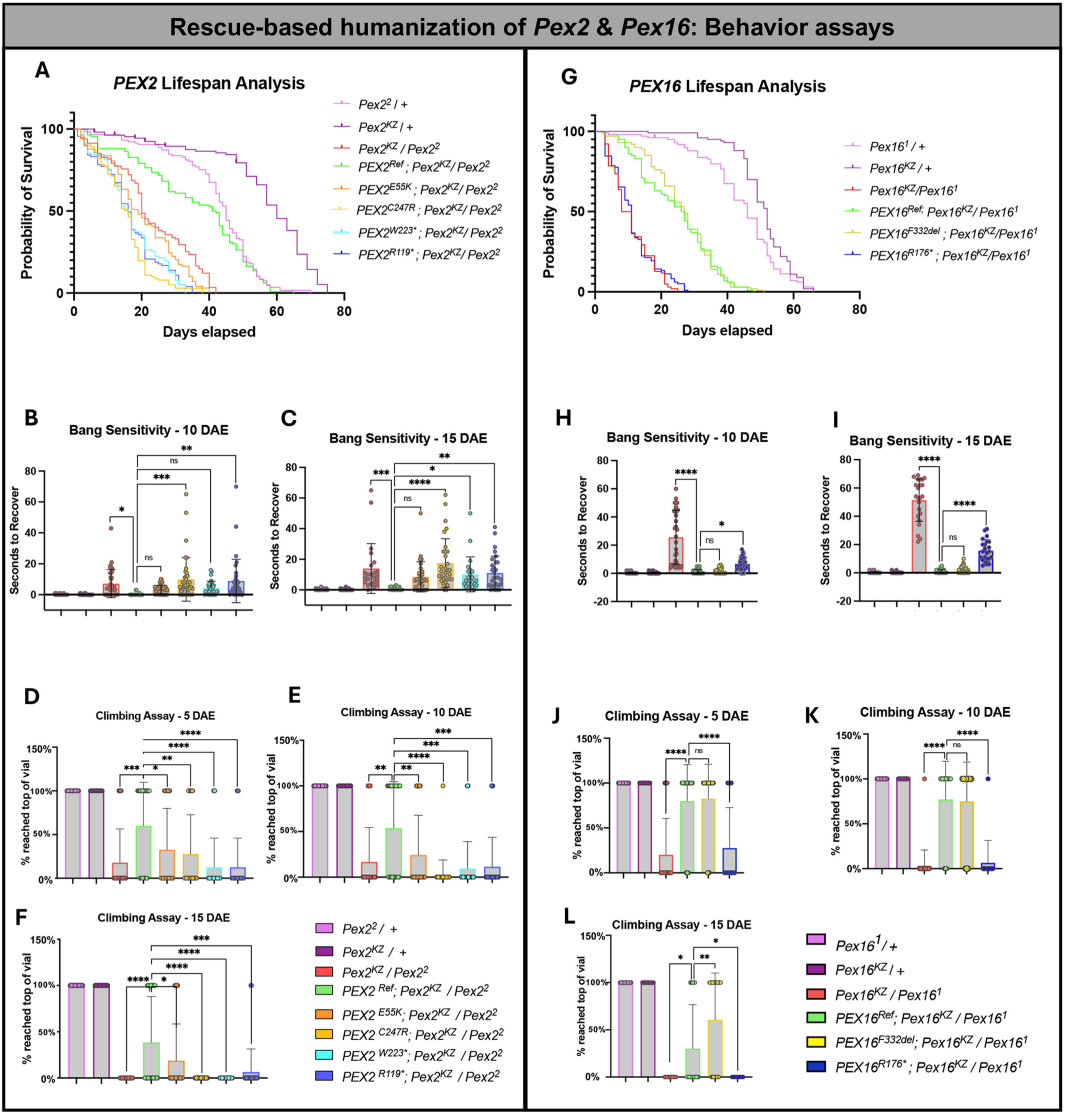
**Rescue-based humanization of *Pex2* and *Pex16* behavior assays.** (A) Lifespan analysis of *PEX2^Ref and Variant^* flies (*n*=110 *PEX2^Ref^*, *n*=98 *PEX2^E55K^*, *n*=101 *PEX2^C247R^*, *n*=102 PEX2^W223^*, *n*=101 PEX2^R119^*) along with our *Pex2* null (*Pex2^KZ^/Pex2^2^ n*=115) and *Pex2* control lines (*n*=117 *Pex2*^*2*^*/*+ and *n*=98 *Pex2*^*KZ*^/+). (B) Bang-sensitivity assay at 10 days after eclosion (DAE) of *PEX2^Ref and Variant^* flies (*n*=26 *PEX2^Ref^*, *n*=37 *PEX2^E55K^*, *n*=38 *PEX2^C247R^*, *n*=32 PEX2^W223^*, *n*=34 PEX2^R119^*), along with *Pex2*^*KZ*^*/Pex2*^*2*^ (*n*=36) and *Pex2* control lines (*n*=40 *Pex2*^*2*^*/*+ and *n*=40 *Pex2*^*KZ*^/+). (C) Bang-sensitivity assay of *PEX2^Ref and Variant^* flies at 15 DAE (*n*=26 *PEX2^Ref^*, *n*=32 *PEX2^E55K^*, *n*=32 *PEX2^C247R^*, *n*=22 PEX2^W223^*, *n*=31 PEX2^R119^*), along with *Pex2*^*KZ*^*/Pex2*^*2*^ (*n*=23 and *Pex2* control lines (*n*=40 *Pex2*^*2*^*/*+ and *n*=40 *Pex2*^*KZ*^/+). (D) Climbing assay at 5 DAE of *PEX2^Ref and Variant^* flies with data indicating if flies either had the ability to climb or not (*n*=30 *PEX2^Ref^*, *n*=40 *PEX2^E55K^*, *n*=40 *PEX2^C247R^*, *n*=40 PEX2^W223^*, *n*=40 PEX2^R119^*), along with *Pex2*^*KZ*^*/Pex2*^*2*^ (*n*=45) and *Pex2* control lines (*n*=40 *Pex2*^*2*^*/*+ and *n*=40 *Pex2*^*KZ*^/+). (E) Climbing assay at 10 DAE of *PEX2^Ref and Variant^* flies (*n*=26 *PEX2^Ref^*, *n*=37 *PEX2^E55K^*, *n*=38 *PEX2^C247R^*, *n*=32 PEX2^W223^*, *n*=34 PEX2^R119^*), along with *Pex2*^*KZ*^*/Pex2*^*2*^ (*n*=36) and *Pex2* control lines (*n*=40 *Pex2*^*2*^*/*+ and *n*=40 *Pex2*^*KZ*^/+). (F) Climbing assay at 15 DAE of *PEX2^Ref and Variant^* flies (*n*=26 *PEX2^Ref^*, *n*=32 *PEX2^E55K^*, *n*=32 *PEX2^C247R^*, *n*=22 PEX2^W223^*, *n*=31 PEX2^R119^*), along with *Pex2*^*KZ*^*/Pex2*^*2*^ (*n*=23) and *Pex2* control lines (*n*=40 *Pex2*^*2*^*/*+ and *n*=40 *Pex2*^*KZ*^/+). (G) Lifespan analysis of *PEX16^Ref and Variant^* flies (*n*=100 *PEX16^Ref^*, *n*=100 *PEX16^F332del^*, *n*=98 PEX16^R176^*), along with our *Pex16* null *Pex16*^*KZ*^*/Pex16*^*1*^ (*n*=102) and *Pex16* control lines (*n*=100 *Pex16*^*1*^*/+* and *n*=100 *Pex16*^*KZ*^*/+*). (H) Bang-sensitivity assay at 10 DAE of *PEX16^Ref and Variant^* flies (*n*=35 *PEX16^Ref^*, *n*=40 *PEX16^F332del^*, *n*=31 PEX16^R176^*), along with *Pex16*^*KZ*^*/Pex16*^*1*^ *n*=32 and *Pex16* control lines (*n*=40 *Pex16*^*1*^*/+* and *n*=40 *Pex16*^*KZ*^*/+*). (I) Bang-sensitivity assay at 15 DAE of *PEX16^Ref and Variant^* flies (*n*=30 *PEX16^Ref^*, *n*=38 *PEX16^F332del^*, *n*=23 PEX16^R176^*), along with *Pex16*^*KZ*^*/Pex16*^*1*^ (*n*=23) and *Pex16* control lines (*n*=40 *Pex16*^*1*^*/+* and *n*=40 *Pex16*^*KZ*^/*+*). (J) Climbing assay at 5 DAE of *PEX16^Ref and Variant^* flies with data indicating if flies either had the ability to climb or not (*n*=40 *PEX16^Ref^*, *n*=40 *PEX16^F332del^*, *n*=40 PEX16^R176^*), along with *Pex16*^*KZ*^*/Pex16*^*1*^ (*n*=40) and *Pex16* control lines (*n*=40 *Pex16*^*1*^*/+* and *n*=40 *Pex16*^*KZ*^*/+*). (K) Climbing assay at 10 DAE of *PEX16^Ref and Variant^* flies (*n*=35 *PEX16^Ref^*, *n*=40 *PEX16^F332del^*, *n*=31 PEX16^R176^*), along with *Pex16*^*KZ*^*/Pex16*^*1*^ (*n*=32) and *Pex16* control lines (*n*=40 *Pex16*^*1*^*/+* and *n*=40 *Pex16*^*KZ*^*/+*)*.* (L) Climbing assay at 15 DAE of *PEX16^Ref and Variant^* flies (*n*=30 *PEX16^Ref^*, *n*=38 *PEX16^F332del^*, *n*=23 PEX16^R176^*), along with *Pex16*^*KZ*^*/Pex16*^*1*^ (*n*=23) and *Pex16* control lines (*n*=40 *Pex16*^*1*^*/+* and *n*=40 *Pex16*^*KZ*^*/+*). *P<0.05, **P<0.01, ***P<0.001, ****P<0.0001; ns, not significant.

To further study the effects of rescue-based humanization of *Pex* genes, we moved to three behavior assays (lifespan, bang sensitivity and climbing) to observe and compare the behavior of the *PEX* variants and reference lines. We performed a lifespan analysis of our humanized *PEX2* variants and reference flies (Fig. 5A). We noted that human *PEX2^Ref^* partially rescues the shortened lifespan of *Pex2^KZ^/Pex2^2^*, and that the variants have a similar lifespan to the *Pex2^KZ^/Pex2^2^* mutant and fail to rescue (Fig. 5A). We performed the Log-rank (Mantel−Cox) test to compare each survival curve to the human *PEX2^Ref^* and the *Pex2* mutant and interpret the significance of rescue in lifespan analysis (Table S2A). We also took note of the *PEX2* sex-specific differences in lifespan (Table S2A; Fig. S1A,B).

Next, we observed the *PEX2* variants and fly reference behavior in the bang-sensitivity assay at 10 and 15 DAE (Fig. 5B,C). On day 10, we found that *PEX2^Ref^* can partially rescue the *Pex2* null phenotype. However, variants PEX2^R119^* and *PEX2^C247R^* fail to rescue, and variants *PEX2^E55K^* and PEX2^W223^* did not differ significantly from the reference (Fig. 5B). At day 15, the flies become slightly more bang-sensitive, showing that there is progressive neuronal dysfunction (Fig. 5C). Interestingly, *PEX2^E55K^* continued to behave not significantly different to *PEX2^Ref^* at day 15 (Fig. 5C). Sex-specific differences in bang-sensitivity were also noted (Fig. S1C,D). We also observed age-dependent worsening of phenotype when observing the ability of flies to climb at 5, 10 and 15 DAE (Fig. 5D-F). Additionally, we took note of differences in climbing behavior between *PEX2* male and female flies (Fig. S1G-L). We found, again, that *PEX2^Ref^* flies were able to partially rescue the fly null phenotype, and that the variants behave similarly to *Pex2* null flies. When expressed in flies, variants PEX2^R119^*, PEX2^C247R^ and PEX2^W223^* all behave as null alleles in each of the above three assays. However, we found that the *PEX2^E55K^* variant – that is associated with mild PBD-ZSD – rescues bang sensitivity and intermediately rescues climbing at different ages. Altogether, the *PEX2^E55K^* allele is likely to be a hypomorphic allele.

We then conducted the same three behavior assays for the *PEX16* variants and reference lines. We first performed a lifespan analysis of our humanized *PEX16* variants and reference flies (Fig. 5G). We noted that the human *PEX16^Ref^* and *PEX16^F332del^* variants partially rescue the shortened lifespan of *Pex16^KZ^/* and that the severe loss-of-function PEX16^R176^* variant has a lifespan similar to that of *Pex16^KZ^/Pex16^1^* (Fig. 5G). We performed the Log-rank (Mantel−Cox) test to compare each survival curve to that of human *PEX16^Ref^* and of *Pex16* mutant and interpret the significance of rescue in lifespan analysis (Table S2B). We also took note of the lifespan differences between *PEX16* male and female flies (Table S2B; Fig. S2A,B). Next, we performed the bang-sensitivity assay on our *PEX16* variant and reference flies at 10 and 15 DAE (Fig. 5H,I). We found that our human reference flies could rescue the *Pex16* null bang sensitivity. Interestingly, the PEX16^R176^* variant flies could not rescue the bang-sensitive phenotype but *PEX16^F332del^* variant flies did not behave significantly different compared to reference flies at day 10 or day 15, and could also rescue the phenotype. Sex-specific differences in bang sensitivity were also noted (Fig. S2C-F). We also observed the ability of *PEX16^Ref^* and variant flies to climb at 5 days, 10 days and 15 days (Fig. 5J-L). We found that the PEX16^R176^* variant behaved similarly to the *Pex16* null flies and did not rescue the phenotype. However, reference and *PEX16^F332del^* flies could partially rescue the climbing defect. Interestingly, the *PEX16^F332del^* flies displayed similar climbing ability as the *PEX16^Ref^* flies at 5 and 10 days but outperformed *PEX16^Ref^* flies at day 15 (Fig. 5L). Additionally, we noticed differences in climbing behavior between *PEX16* male and female flies **(**Fig. S2G-L). In summary, the *PEX16^F332del^* allele, which is associated with an atypical clinical presentation of ataxia, exhibits complete rescue of shortened lifespan, complete rescue of bang sensitivity at days 10 and 15, and overall better rescue than the reference for climbing ability.

### Loss of *Pex* genes leads to phenotypes in the direct flight muscle, but the human *PEX* reference is able to rescue

**Fig. 6.**
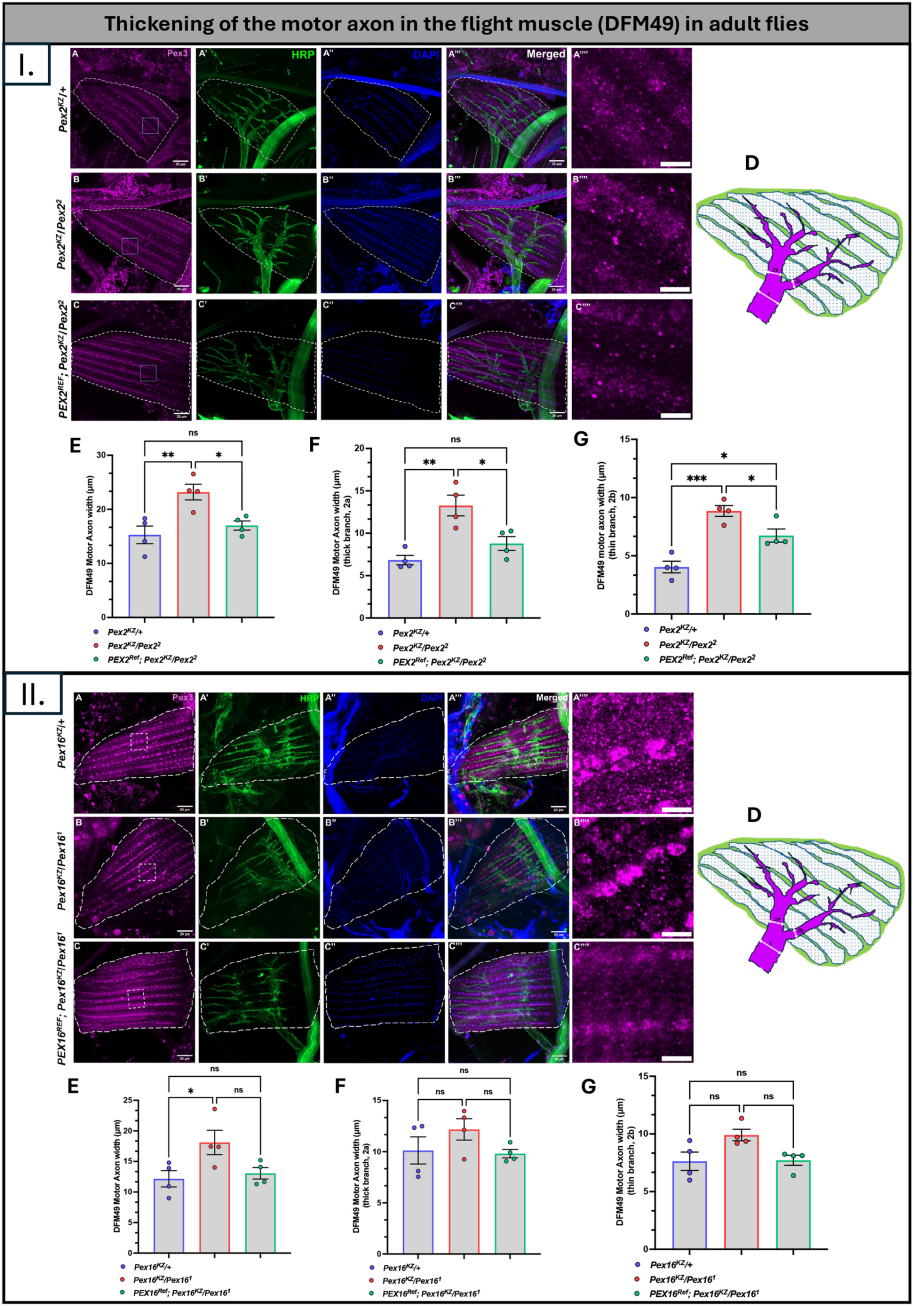
**Thickening of the motor axon within DFM49 of adult flies.** (I, top) Panels A-A‴ represent *Pex2* control group flies (*Pex2^KZ^/+*); panels B-B‴ represent *Pex2* null flies (*Pex2^KZ^/Pex2^2^*) and panels C-C‴ represent human *PEX2* rescue group flies (*PEX2^Ref^*;*Pex2^KZ^/Pex2^2^*). Dashed lined surround the DFM49. Panels A-C show staining for peroxisomes (Pex3, purple), panels A′-C′ show staining for nerve fiber tracks (HRP, green). Panels A″-C″ show nuclei (blue) stained with DAPI. Boxed areas in A-C are shown magnified in A″″-C″″, respectively. (D) Schematic representation of the DFM49, with numbers within indicating the motor axon entering the flight muscle (1), the thick branch (2a) and the thin branch (2b). (E) Quantification of the *Pex2* DFM49 motor axon width between the three genotypes. (F) Quantification of the thick branch of the *Pex2* DFM49 motor axon width between the three genotypes. (G) Quantification of the thin branch of the *Pex2* DFM49 motor axon width between the three genotypes. *n*=4 *Pex2^KZ^/+* control, *n*=4 *Pex2^KZ^/Pex2^2^*, *n*=4 *PEX2^Ref^*. (II, bottom) Panels A-A‴ represent *Pex16* control group flies (*Pex16^KZ^/+*); panels B-B‴ represent *Pex16* null flies (*Pex16^KZ^/Pex16^2^*) and panels C-C‴ represent human *PEX16* rescue group flies (*PEX16^Ref^*;*Pex16^KZ^/Pex16^2^*). Dashed lined surround the DFM49. Panels A-C show staining for peroxisomes (Pex3, purple), panels A′-C′ show staining for nerve fiber tracts (HRP, green). Panels A″-C″ show nuclei (blue) stained with DAPI. Boxed areas in A-C are shown magnified in  A″″-C″″, respectively. (D) Schematic representation of the DFM49, with numbers within indicating the motor axon entering the flight muscle (1), the thick branch (2a) and the thin branch (2b). (E) Quantification of *Pex16* DFM49 motor axon width between the three genotypes. (F) Quantification of the thick branch of the *Pex16* DFM49 motor axon width between the three genotypes. (G) Quantification of the thin branch of the *Pex16* DFM49 motor axon width between the three genotypes. *n*=4 *Pex16^KZ^/+* control, *n*=4 *Pex16^KZ^/Pex16^1^*, *n*=4 *PEX16^Ref^*. ns, not significant. *P<0.05, **P<0.01, ***P<0.001. Scale bars: 20 µm (A-C‴ in I and II), 5 µm (A″″-C″″ in I and II).

In our previous characterization of *Pex2* and *Pex16* deletion alleles, we observed climbing, lifespan and flight defects (Wangler et al., 2017). Given that we saw locomotor and lifespan rescue with the human transgenes, we decided to assess the morphology of a direct flight muscle (DFM), i.e. DFM49. In control flies (*Pex2^KZ^/+*), we observed Pex3 puncta localized between muscle fibers and stained with horseradish peroxidase (HRP) to examine the innervating nerve fiber in adult 0-day-old flies (Fig. 6I, panel A). In the *Pex2*^*KZ*^*/Pex2^2^* mutant, we observed a reduction in Pex3 staining and an apparent thickening of the surrounding nerve fiber, an intriguing finding since day 10-15 mutant adult flies exhibit locomotor defects in bang-sensitivity and climbing assays (Fig. 6I, panel B). Expression of the human *PEX2* transgene appeared to rescue peroxisomes (Pex3 staining) and the nerve fiber thickening observed in the mutant (Fig. 6I, panel C). We measured the width of the DFM motor axon (base, thick and thin branches) to observe the differences in width, both proximal and distal (Fig. 6I, panel D). We found that the human *PEX2* transgene rescues the thickening of the nerve fiber observed in the mutant (Fig. 6I, panels E-G).

Similarly, we assessed the morphology of DFM49 in *Pex16* flies. In our control flies, we observed Pex3 puncta intercalating the muscle fiber and HRP innervating the nerve fiber in 0-day-old flies (Fig. 6II, panel A). In our *Pex16* mutant, we observed a dramatic reduction in Pex3 staining and thickening of the innervating nerve fiber (Fig. 6II, panel B). However, the human *PEX16* transgene expression is not able to fully rescue the nerve fiber thickening seen in the *Pex16* mutant (Fig. 6II panels C, E-G). We also assessed the morphology of DFM49 in *PEX2* mild and variants (Fig. S3A-F). We found that the human variants were able to partially rescue the thickening of the motor axon that was observed in the *Pex2* mutant, that the *PEX2^C247R^* variant exhibits slight thickening in the distal motor branches, and that the *PEX2^E55K^* variant exhibits slight thickening in the proximal motor branch.

## DISCUSSION

In this study, we generated humanized *Drosophila Pex2* and *Pex16* to study evolutionary conservation of *PEX* genes across species, and to assay the effect of human mutations (Bellen and Yamamoto, 2015; Harnish et al., 2019). Therefore, we generated *KozakGAL4* alleles for which we replaced the coding sequence of the fly gene with *GAL4*, generating a driver that allows expression of the human gene instead (Kanca et al., 2022). Upon finding that these unique alleles effectively serve as null mutants, we used the *KozakGAL4* lines to express human UAS-cDNAs of our targeted *PEX2* and *PEX16* gene and variants to assess rescue of the fly null phenotype. We began testing the impact of human proteins and their ability to rescue peroxisomal morphology defects in *Drosophila*. We found that *Pex2* and *Pex16* null mutants have a significantly reduced number of Pex3 puncta, but that *PEX2^Ref^* and *PEX16^Ref^* can partially rescue these numbers, demonstrating that human proteins can partially function in place of the fly protein *in vivo*. One factor to consider is that the human transgenes were tagged with GFP. With these findings, *PEX2* and *PEX16* human reference constructs successfully restore peroxisomes, confirming the generation of humanized flies for *PEX2* and *PEX16*. It is remarkable to note that, for these two *Pex* genes, the human proteins can participate in fly peroxisome biogenesis in distinct steps of the process, despite over 500 million years of evolution separating the fly and human genome.

In addition to humanization, another benefit of the *KozakGAL4* lines for *Pex2* and *Pex16* is that we were able to see the endogenous expression pattern of these genes in the fly. While we would expect peroxisome biogenesis to be a general process, in this study, we did not observe ubiquitous expression of *Pex2* or *Pex16*, and there were differences in *Pex16* and *Pex2* expression patterns. This could reflect differences during development, although we were able to see differences in both larvae and adult brains. For all such tests of gene expression by using insertion alleles and testing what approximates an enhancer trap, it is possible that the expression pattern does not fully recapitulate all the enhancers of the endogenous gene. However, the insertions were at the *Pex2* and *Pex16* locus, respectively, and we also showed significant rescue with human transgenes when using these same fly lines, indicating the sufficiency of the enhancers trapped by the *Kozak-GAL4* line. Additionally, *Pex2* and *Pex16* are expressed in diverse cells according to single-cell RNA sequencing data (Kanca et al., 2022). Therefore, our conclusion is consistent with other datasets, suggesting that *Pex2* and *Pex16* are not always expressed in the same cell populations.

By performing various behavior assays, we found that *PEX2^Ref^* and *PEX16^Ref^* were able to improve the survival phenotype in *Pex2*- and *Pex16*-deficient models, as well as to restore bang sensitivity and defective climbing behavior of the flies. Interestingly, in addition to confirming human rescue, we also found that PBD variants that have previously been characterized as mild or atypical also produced a mild phenotype in the various assays. After utilizing rescue-based humanization of *Pex* genes, we found that our *PEX2^E55K^* and *PEX16^F332del^* variants display milder behavior phenotypes, compared to the other pathogenic variants. This finding corresponds to previous studies on mild PBD variants (Imamura et al., 1998; Shimozawa et al., 1999; Fujiwara et al., 2000; Bacino et al., 2015a,b), suggesting an allelic spectrum for *PEX2* and *PEX16*.

Based on previous studies, the four *PEX2* variants chosen by us show a range in clinical severity for classic PBD-ZSD (Shimozawa et al., 1992, 1999; Mandel et al., 1994; Imamura et al., 1998; Fujiwara et al., 2000; Gootjes et al., 2004; Mignarri et al., 2012). The *PEX2^E55K^* variant has been identified in patients with mild PBD-ZSD in compound heterozygosity with the PEX2^R119^* variant (Imamura et al., 1998; Shimozawa et al., 1999; Fujiwara et al., 2000). In our assays, we found that the *PEX2^E55K^* variant has a less-severe phenotype compared to the other *PEX2* variants. It performed best in lifespan analysis compared to the other variants, showed the least-severe bang-sensitive phenotype as well as a only slight decrease in climbing ability and was significantly better than the other variants. *PEX2^E55K^* never fully rescued the phenotypes and, therefore, consistently was a hypomorph. Our other three *PEX2* variants have been characterized as being pathogenic but the clinical significance in patients varies. The homozygous PEX2^W223^* variant has been identified in a patient with mild-PBD-ZSD (Mandel et al., 1994; Gootjes et al., 2004). Although PEX2^W223^* flies did not perform as well in the various assays, it showed slightly better performance in viability and bang-sensitivity assays than the PEX2^R119^* and *PEX2^C247R^* variants. The PEX2^R119^* variant has been identified in patients with severe as well as milder cases of PBD-ZSD, with homozygous probands showing a trend towards a more-severe manifestation of the disease, and patients with a compound heterozygous genotype showing milder or atypical presentations (Shimozawa et al., 1992, 1999; Gootjes et al., 2004; Mignarri et al., 2012). In flies, PEX2^R119^* had a more-severe phenotype in behavior assays than the *PEX2*^*E55K*^ and *PEX2*^W223^* variants. Last, the presence of the homozygous *PEX2^C247R^* variant is also associated with a severe PBD-ZSD phenotype (Gootjes et al., 2004). In flies, *PEX2^C247R^* also displayed the overall most-severe phenotype in the behavior assays. With these findings, we propose an allelic severity spectrum, in which *PEX2^E55K^* is the least-severe and *PEX2^C247R^* the most-severe variant (*PEX2^E55K^*<PEX2^W223^*<PEX2^R119^*<*PEX2^C247R^*) (Table 1).

**Table 1.** Clinical and functional severity of *PEX2* and *PEX16* gene mutations

	**Consequence**	**Clinical significance**	**Clinical severity**	**Observed outcome**
***PEX2* variant**				
E55K	Missense	Probably pathogenic	Mild PBD-ZSD in compound heterozygosity with R119*	Viable F1 progeny Higher lifespan compared to other variants First less-severe bang sensitivity Slight climbing ability
W223*	Nonsense	Pathogenic	Mild PBD-ZSD in homozygosity	Semi-viable F1 progeny Second less-severe bang sensitivity Decreased lifespan Severe climbing defect
R119*	Nonsense	Pathogenic	Severe PBD-ZSD in homozygosity Mild PBD-ZSD in compound heterozygosity with E55K Atypical PBD-ZSD in compound heterozygosity with 1-bp insertion in PEX2	Semi-viable F1 progeny Third less-severe bang-sensitivity Decreased lifespan Severe climbing defect
C247R	Missense	Pathogenic	Severe PBD-ZSD in homozygosity	Semi-viable F1 progeny Most-severe bang-sensitivity Decreased lifespan Severe climbing defect
				
***PEX16* variant**				
F332del	In-frame deletion	Probably pathogenic	Atypical PBD-ZSD in homozygosity	Viable F1 progeny Higher lifespan Less-severe bang-sensitivity Better climbing ability than *PEX16* reference
R176*	Nonsense	Pathogenic	Severe PBD-ZSD in homozygosity	Semi-viable F1 progeny Decreased lifespan Severe bang-sensitivity Severe climbing defect

Table 1 lists the severity of human PEX2 and PEX16 variants, considering clinical significance and disease severity, as well as outcomes observed in this study.

We also found an allelic spectrum for our two *PEX16* variants. The *PEX16^F332del^* variant has previously been reported by us in a patient homozygous for the variant, showed an atypical presentation of PBD-ZSD – i.e. as having ataxia but normal peroxisomal biochemical testing in plasma (Bacino et al., 2015a,b). In flies, we found that *PEX16^F332del^* had completely viable F1 progeny, a lifespan similar to that of *PEX16^Ref^*, could rescue bang sensitivity and even outperformed the human reference in the climbing assay at day 15. Alternatively, the homozygous PEX16^R176^* variant has been described in a patient, who presented with a severe PBD-ZSD phenotype (Honsho et al., 1998; Ohba et al., 2013). In flies, we observed PEX16^R176^* to result in a much more-severe behavior phenotype that would be consistent with a null allele. PEX16^R176^* flies showed semi-viability regarding their F1 progeny, decreased lifespan, severe bang sensitivity and a severe climbing defect. With these findings, we propose an allelic severity spectrum, with *PEX16^F332del^* being a mild and PEX16^R176^* a severe variant (*PEX16^F332del^*<PEX16^R176^*) (Table 1).

Our data are informative for understanding the difference between mild PBD-ZSD with classic involvement of the retina and hearing versus atypical ataxia phenotypes. The *PEX16^F332del^* variant is associated with atypical ataxia with normal retinal and hearing findings (Bacino et al., 2015a,b). This atypical ataxia phenotype for *PEX16* has been characterized by other groups but lacks the classic features of PBD-ZSD, and its relationship to the classic spectrum has been unclear (Ebberink et al., 2010; Cheung et al., 2022). Our data show that the ataxia allele functioned as a weak hypomorphic allele in certain contexts, while exhibiting wild-type or enhanced activity relative to the reference allele in others, including the climbing assay used to assess negative geotaxis. This suggests that a certain expression level of very mild *PEX16* alleles can produce unique phenotypes that are not clinically recognizable as PBD-ZSD.

Severe alleles in *PEX* genes, including *PEX2^C247R^* and PEX16^R176^*, lead to severe clinical presentations; however, in recent years, mild clinical presentations due to *PEX* mutations have expanded from phenotypes referred to as infantile Refsum disease that includes atypical ataxia in the *PEX16*^*F332del*^ variant, and Heimler syndrome associated with variants of *PEX1* and *PEX6* (Rosewich et al., 2005; Waterham and Ebberink, 2012; Bacino et al., 2015a,b). How these mild conditions can differ so drastically is not yet understood. The use of humanized flies to continue building an allelic spectrum as additional variants emerge has the great potential to fully distinguish the differences between these alleles. In the future, it will be interesting to determine how pathogenic variants, such as *PEX1* and *PEX6* variants, involved in Heimler syndrome, alter the behavior and peroxisomal morphology in *Drosophila* compared to the *PEX2* and *PEX16* variants studied here. The tools we developed will be useful for future pharmacologic and genetic screens targeting milder *PEX* variants. The use of *Drosophila* allows multiple *in vivo* assays for these variants to probe the pleiotropic effects of *PEX* genes on the neurological system.

## MATERIALS AND METHODS

### Fly strains and maintenance

All flies were maintained at room temperature (21°C), except when otherwise noted. Experiments were conducted at room temperature.

### *Pex2* lines

*TI{KozakGAL4} Pex2 [CR70193-KO-kG4]* (here referred to as *Pex2^KZ^*) [Bloomington Drosophila Stock Center (BDSC) #94997] were generated as described by Kanca et al. (2022). sgRNA target sites in *Pex2* UTRs are as 5′-TTTGTTTATATTCTTGCCTTTGG and GGTTCTGCGTGTCCTGAGTCGGG-3′. Information about homology arms and the primers used to verify the insertions can be found at https://flypush.research.bcm.edu/pscreen/crimic/crimic.php.

The *Pex2^1^* and *Pex2^2^* lines were derived from imprecise excision of (*w[1118]; P{w[+mC]=EPg}pex2[HP35039]/TM3,Sb[1]*. These lines were repeatedly crossed (i.e. backcrossed) for five generations with *yw:FRT80B* to ensure experimental consistency, and studied as (a) yw; FRT80B-Pex2^1^ (here referred to as Pex2^1^), (b) yw; FRT80B-Pex2^2^ (here referred to as Pex2^2^) and (c) w[1118];PBac{y[+mDint2] w[+mC]=53M21}VK00037; FRT80B- Pex2^2^ (here referred to as Pex2^2^ Rescue). Additionally, a genomic deficiency line uncovering the *Pex2* locus, i.e. w118;Df(3L)BSC376/TM6C,Sb1 (here referred to as *Pex2^Df^*) was used.

For *Pex2* excisions, *w[1118]; P{w[+mC]=EPg}Pex2[HP35039]/TM3, Sb[1]* was crossed to *yw; CyO*, *delta2-3/Egf*r and progeny crossed to *y w; D/TM6B*, *Tb*. Progeny were screened for loss of *w+* marker. Using PCR, 461 independent excision lines were screened; selected were *pex2-1* – a 7-bp insertion within a 606 bp deletion, and *pex2-2* – an 11-bp insertion within a 473 bp deletion.

For the *Pex2^2^* rescue strain, CH322-53M21 from the CHORI-322_BAC P[acman] genomic clone collection was obtained for a *Pex2* genomic clone. The plasmid was amplified and grown, then purified and injected into *y[1] w[1118]; PBac{y[+]-attP}VK00037* embryos (VK00037 contains an attP on 2 L at 22A3). Transformants were selected based on the w+ marker and these strains were balanced to generate y[1] w[*]; Dp(3;2)CH322-53M21, PBac{y[+mDint2] w[+mC]=CH322-53M21}VK00037. The line was then crossed into the mutant strains listed above.

For the generation of the human UAS-*PEX2* lines for Reference, E55K, R119X, W223X, C247R we obtained constructs which were codon-optimized for *Drosophila* with a C-terminal GFP tag and encoded the human protein from GeneART™ (Thermo Fisher Scientific) (Fig. S4). These constructs were subcloned into the pUAST-attB vector via NotI and XhoI restriction sites. The UAS-constructs were then injected and inserted into the same genomic locus on the second chromosome (VK37 docking site) via ϕC31-mediated transgenesis (Venken et al., 2006).

### *Pex16* lines

*TI{KozakGAL4} Pex16 [CR70194-KO-kG4]* (here referred to as *Pex16^KZ^*) (BDSC# 94998) were generated as described by Kanca et al. (2022). sgRNA target sites in *Pex16* UTRs were 5′-ATAAAAATAATGAGGTGTTTCGG-3′ and 5′-TAGAGCGTTAGTATTCCCCTAGG-3′. Information about homology arms and the primers used to verify the insertions can be found at https://flypush.research.bcm.edu/pscreen/crimic/crimic.php.

The yw:Pex16^1^ line was obtained from Kenji Matsuno (KYOTO Drosophila Stock Center, Kyoto, Japan), derived from y^1^w^67c23^; P{w[+mC]y[+mDint2]=EPgy2}Pex16 [EY05323], yw; Pex16^1^ (here referred to as Pex16^1^).

For the generation of the human UAS-*PEX16* lines for Reference, R176X and del955TCT (F332del), we obtained constructs that had been codon-optimized for *Drosophila* with a C-terminal GFP tag and encoded the human protein from GeneART™ (Thermo Fisher Scientific) (Fig. S4). These constructs were subcloned into the pUAST-attB vector via NotI and XhoI restriction sites. The UAS-constructs were then injected and inserted into the same genomic locus on the second chromosome (VK37 docking site) via ϕC31-mediated transgenesis (Venken et al., 2006).

### Lifespan determination

Flies were collected under CO_2_ between 1 h and 24 h after eclosion. Male and female flies were separated and kept at 25°C, with 10-15 flies per vial. Of each line, 100 flies (50 females and 50 males) were collected. Fly food was changed every 3 days. The number of live flies was checked every 3 days until the last fly had died. A tally of the number of flies and their lifespan was kept, and data were analyzed with a Kaplan–Meier survival curve.

### Bang-sensitivity assay

Flies were kept without exposure to CO_2_ for at least 48 h prior to the assay. Flies were then individually vortexed in an empty vial for 10 s using a Fisher Scientific Vortex Mixer, and we timed how long it took the individual fly to recover to normal behavior (i.e. no longer paralyzed or flapping wings at high frequency) after being vortexed. Bang sensitivity was done for flies at 5, 10 and 15 DAE.

### Climbing assay

Flies were kept without exposure to CO_2_ for at least 48 h prior to the assay. Climbing assay was performed on flies at 5 days, 10 days, and 15 DAE. Flies were individually placed into an empty vial, tapped to the bottom of the vial, and timed to note how long it took for them to climb to the 8 cm mark at the top of the vial. If the fly did not reach the top of the vial within 60 s, we recorded the fly as not having the ability to climb.

### Immunocytochemistry

Tissue samples were collected from wandering third-instar larvae for body wall muscle and from the adult thorax for direct flight muscle 49 (DFM49). Dissections were performed on Sylgard plates using 1×PBST [1% Tween 20 in 1×phosphate-buffered saline (PBS)]. The dissected larvae were fixed in 4% paraformaldehyde for 10 min at room temperature. After fixation, they were washed three times with PBST for 10 min each and then blocked with goat serum for 30 min.

Samples were incubated overnight at 4°C with primary antibodies, followed by three additional washes with PBST. Next, they were incubated overnight at 4°C with secondary antibodies and washed again three times with PBST. Finally, the samples were mounted on slides using Vectashield containing DAPI.

The primary antibody used was rabbit anti-Pex3 at a dilution of 1:500, (obtained from the laboratory of James McNew, Rice University, Houston, TX). The secondary antibody was donkey anti-rabbit IgG conjugated to Cy3, obtained from Jackson ImmunoResearch Laboratory (Catalogue no.: 711-165-152) at a dilution of 1:1000. For immunostaining neuromuscular junctions in adult DFM49, we used Alexa Fluor 647-conjugated rabbit anti-horseradish peroxidase (HRP) obtained from Jackson ImmunoResearch Laboratory (catalogue no.: 323-605-021) at a dilution of 1:1000, which specifically recognizes the HRP epitope present on the surface of all *Drosophila* neurons.

### Dissection of direct flight muscle 49

Dissection of the direct flight muscle 49 (DFM49) was performed following a previously published protocol (Xu et al., 2017). Individual flies were pinned on 35-mm Petri-dish plates filled with Sylgard 184 (Electron Microscopy Sciences) in PBS. A tungsten needle (Fine Science Tools, Foster City, CA, catalog #10130-05) was used to puncture one side of the thoracic cuticle surrounding the wing hinge, which was cut with micro scissors and removed from the remainder of the body. This cuticle piece was transferred to a fresh plate and cleaned by fine forceps to remove any indirect flight muscles and other tissue to expose the set of DFMs, including DFM49. The cuticle piece with DFM49 was then washed in PBS, fixed in 4% PFA for 10 min followed by three washes for 5 min each with PBST (PBS with 0.5% Tween-20). The tissue was then processed for immunostaining.

### Fluorescence microscopy imaging

Imaging was performed using a Zeiss LSM800 Airyscan confocal microscope. Images were acquired with a Plan-Apochromat 40×/1.2 NA water immersion objective, using a frame size of 1024×1024 pixels. The excitation and emission wavelengths were as follows: DAPI: ex405/em410-480 nm; AF488: ex488/em493-570 nm and ex561/em576-700 nm. Raw images were processed using the Airyscan Processing module in Zen 2.6 – Blue edition (Carl Zeiss Microscopy GmbH, Germany), applying the 2D SR processing option. The Airyscan filtering, utilizing a Wiener filter for deconvolution, was set to ‘standard’.

### Image quantitative analysis

Data of all fluorescence images displaying anti-Pex3 immunostaining were quantified and analyzed using the Surface module of Imaris v9.8.2 (Bitplane, Zurich, Switzerland). In this module, surfaces were generated for the fluorophore signal of interest, employing the background-subtraction option. Further, puncta were collected using area filters and to restrict background noise, the optimized lower limit was set to 0.5 µm^2^, surface areas of ≥0.5 µm^2^ were collected for further processing. This threshold approach helped to reduce background noise and minimize false positives during surface creation. The nuclear signal was not included in the quantitation. To avoid nuclear areas, the surface masking approach was used from IMARIS surface module. To do this the surface of a nucleus was created and masked on the Pex3 surface, with voxels inside the nuclear surface set to zero, thereby neglecting all Pex3 signals located within nuclei. The number of surfaces created represented that of individual puncta and was obtained from statistical data generated for the created surface area.

For DFM49, the axon thickness was obtained using Image J, where the widths of axon terminal main and secondary branches were calculated and the obtained data compared among genotypes.

### Statistical analysis

Statistical analysis was completed using GraphPad Prism (Version 10.1.1). Continuous analysis was completed using Ordinary one-way ANOVA with multiple comparisons test (Dunnett's test), where differences between groups were quantified and a *P*-value <0.05 was considered significant. To analyze survival data, we used GraphPad Prism (Version 10.1.1) to graph Kaplan–Meier survival curves. To compare the survival curves, we utilized the Log-rank (Mantel−Cox) test, which determines if two or more Kaplan–Meier curves are significantly different based on assumptions of the Kaplan–Meier analysis, and includes non-informative censoring and proportional hazards.

## Supplementary Material

10.1242/dmm.052258_sup1 Supplementary information

Table S1. *Drosophila Pex2* & *Pex16* survival curve comparison test. (**A**) Indicates the results of the Log-rank test survival curve comparison tests of both female and male *Pex2* lifespan. (**B**) Indicates the results of the Log-rank test survival curve comparison tests of both female and male *Pex16* lifespan. [* = p-value is less than 0.05. ** = p-value is less than 0.01. *** = p-value is less than 0.001. **** = p-value is less than 0.0001]

Table S2. Human *PEX2* & *PEX16* survival curve comparison test. (**A**) Indicates the results of the Log-rank test tests of the *PEX2* (male and female), female only *PEX2*, and male only *PEX2* survival curves between all genotypes. (**B**) Indicates the results of the Log-rank test tests of the *PEX16* (male and female), female only *PEX16*, and male only *PEX16* survival curves between all genotypes. [* = p-value is less than 0.05. ** = p-value is less than 0.01. *** = p-value is less than 0.001. **** = p-value is less than 0.0001]

Table S3. Observed/expected Mendelian ratio calculation of the F1 generation of human *PEX2* and *PEX16* crosses.
