# Supplementary material for: Distinguishing PEX2 and PEX16 gene variant severity for mild, severe and atypical peroxisome biogenesis disorders
Source: Dis Model Mech. 2025 Jul 28;18(7):dmm052258. doi: 10.1242/dmm.052258 (PMC12352285; doi:10.1242/dmm.052258)
Supplement: Supplementary information [file dmm-18-052258-s1.pdf]

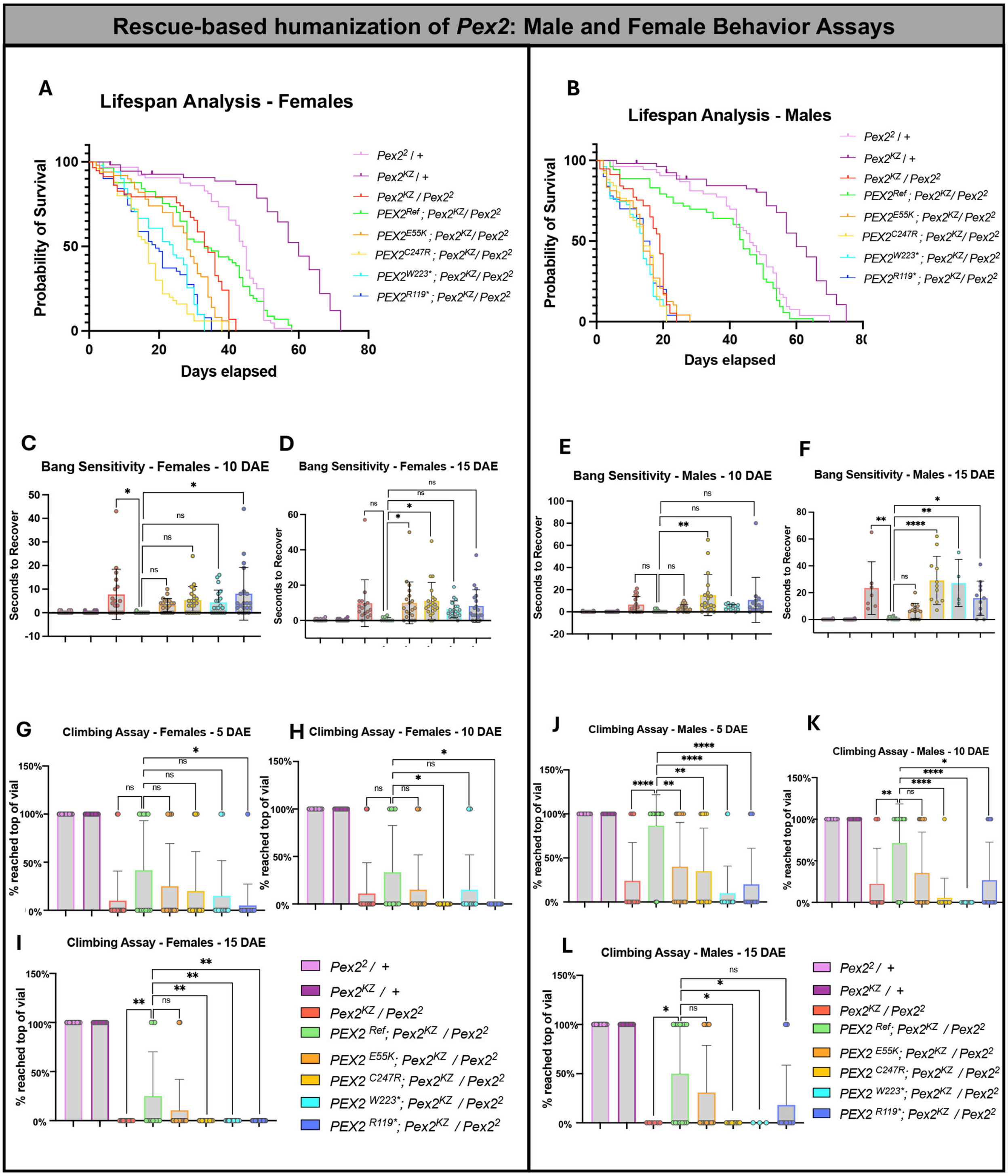

Disease Models & Mechanisms • Supplementary information

Fig. S1. Rescue-based humanization of *Pex2*: Male and female behavior assays.

(**A**) Lifespan analysis of *PEX2$^{Ref\ and\ Variant}$* female flies (n=57 *PEX2^{Ref}*, n=50 *PEX2^{E55K}*, n=50 *PEX2^{C247R}*, n=51 *PEX2^{W223*}*, n=51 *PEX2^{R119*}*) along with our *Pex2* null (n=58) and control lines (pink n=64 and purple n=50). (**B**) Lifespan analysis of *PEX2$^{Ref\ and\ Variant}$* male flies (n=53 *PEX2^{Ref}*, n=48 *PEX2^{E55K}*, n=51 *PEX2^{C247R}*, n=51 *PEX2^{W223*}*, n=50 *PEX2^{R119*}*), along with our *Pex2* null (n=57) and control lines (pink n=53 and purple n=48).

(**C**) Bang sensitivity assay at 10 DAE of *PEX2$^{Ref\ and\ Variant}$* female flies (n=12 *PEX2^{Ref}*, n=20 *PEX2^{E55K}*, n=20 *PEX2^{C247R}*, n=20 *PEX2^{W223*}*, n=20 *PEX2^{R119*}*), along with our *Pex2* null (n= 18) and control lines (pink n=20 and purple n=20). (**D**) Bang sensitivity assay of *PEX2$^{Ref\ and\ Variant}$* female flies at 15 DAE (n=12 *PEX2^{Ref}*, n=19 *PEX2^{E55K}*, n=20 *PEX2^{C247R}*, n=18 *PEX2^{W223*}*, n=19 *PEX2^{R119*}*), along with our *Pex2* null (n= 16) and control lines (pink n=20 and purple n=20). (**E**) Bang sensitivity assay at 10 DAE of *PEX2$^{Ref\ and\ Variant}$* male flies (n=14 *PEX2^{Ref}*, n=17 *PEX2^{E55K}*, n=18 *PEX2^{C247R}*, n=12 *PEX2^{W223*}*, n=14 *PEX2^{R119*}*), along with our *Pex2* null (n= 18) and control lines (pink n=20 and purple n=20). (**F**) Bang sensitivity assay of *PEX2$^{Ref\ and\ Variant}$* male flies at 15 DAE (n=14 *PEX2^{Ref}*, n=13 *PEX2^{E55K}*, n=12 *PEX2^{C247R}*, n=4 *PEX2^{W223*}*, n=12 *PEX2^{R119*}*), along with our *Pex2* null (n= 7) and control lines (pink n=20 and purple n=20). (**G**) Climbing assay at 5 DAE of *PEX2$^{Ref\ and\ Variant}$* female flies (n=12 *PEX2^{Ref}*, n=20 *PEX2^{E55K}*, n=20 *PEX2^{C247R}*, n=20 *PEX2^{W223*}*, n=20 *PEX2^{R119*}*), along with our *Pex2* null (n=20) and control lines (pink n=20 and purple n=20). (**H**) Climbing assay at 10 DAE of *PEX2$^{Ref\ and\ Variant}$* female flies (n=12 *PEX2^{Ref}*, n=20 *PEX2^{E55K}*, n=20 *PEX2^{C247R}*, n=20 *PEX2^{W223*}*, n=20 *PEX2^{R119*}*), along with our *Pex2* null (n= 18) and control lines (pink n=20 and purple n=20). (**I**) Climbing assay at 15 DAE of *PEX2$^{Ref\ and\ Variant}$* female flies (n=12 *PEX2^{Ref}*, n=19 *PEX2^{E55K}*, n=20 *PEX2^{C247R}*, n=18 *PEX2^{W223*}*, n=19 *PEX2^{R119*}*), along with our *Pex2* null (n= 16) and control lines (pink n=20 and purple n=20). (**J**) Climbing assay at 5 DAE of *PEX2$^{Ref\ and\ Variant}$* male flies (n=18 *PEX2^{Ref}*, n=20 *PEX2^{E55K}*, n=20 *PEX2^{C247R}*, n=20 *PEX2^{W223*}*, n=20 *PEX2^{R119*}*), along with our *Pex2* null (n=25) and control lines (pink n=20 and purple n=20). (**K**) Climbing assay at 10 DAE of *PEX2$^{Ref\ and\ Variant}$* male flies (n=14 *PEX2^{Ref}*, n=17 *PEX2^{E55K}*, n=18 *PEX2^{C247R}*, n=12 *PEX2^{W223*}*, n=14 *PEX2^{R119*}*), along with our *Pex2* null (n= 18) and control lines (pink n=20 and purple n=20). (**L**) Climbing assay at 15 DAE of *PEX2$^{Ref\ and\ Variant}$* male flies (n=14 *PEX2^{Ref}*, n=13 *PEX2^{E55K}*, n=12 *PEX2^{C247R}*, n=4 *PEX2^{W223*}*, n=12 *PEX2^{R119*}*), along with our *Pex2* null (n= 7) and control lines (pink n=20 and purple n=20). Ordinary one-way ANOVA with multiple comparisons test.

[* = *p*-value is less than 0.05. ** = *p*-value is less than 0.01. *** = *p*-value is less than 0.001. **** = *p*-value is less than 0.0001]

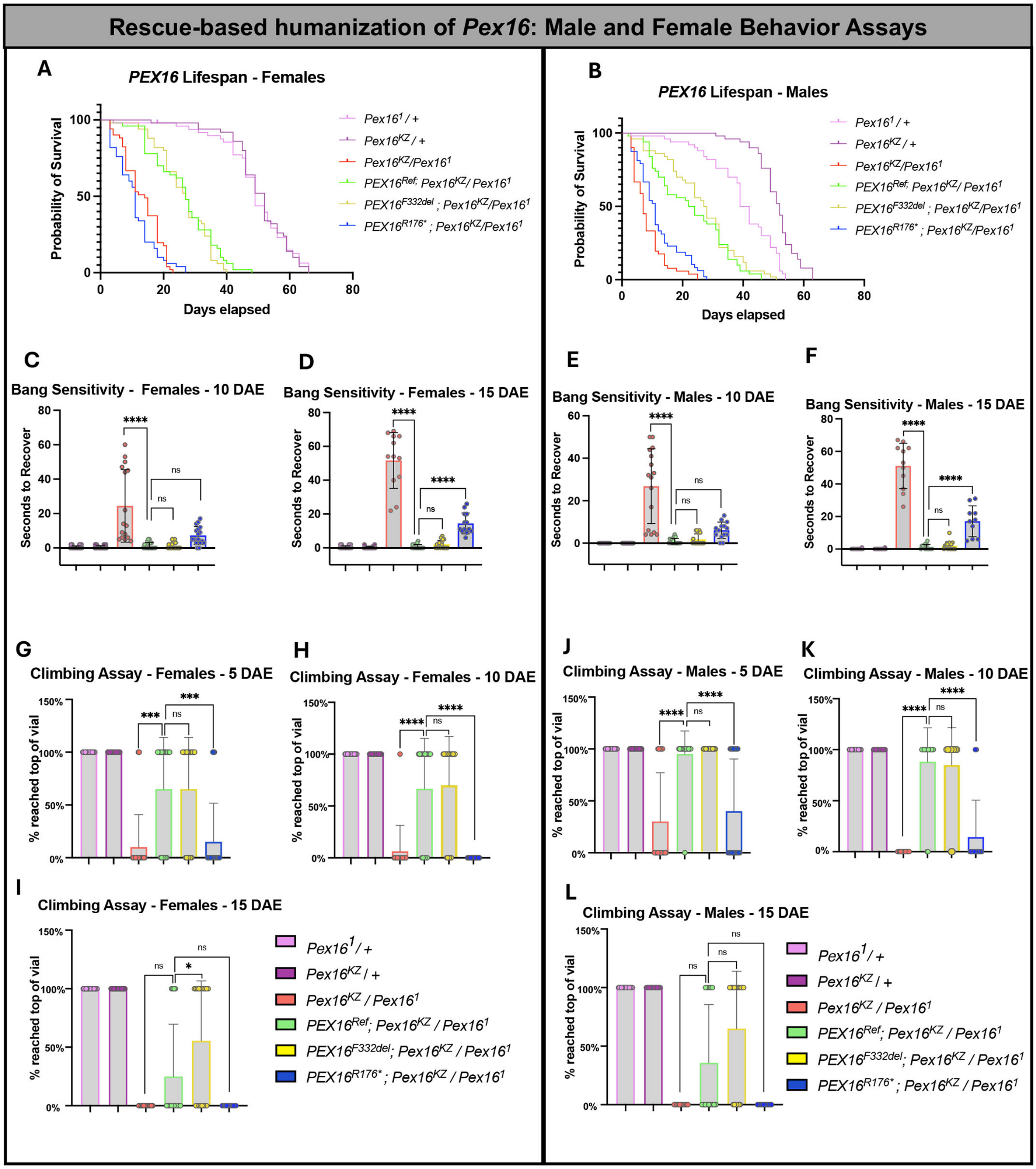

Fig. S2. Rescue-based humanization of *Pex16*: Male and female behavior assays.
(**A**) Lifespan analysis of $PEX16^{Ref\ and\ Variant}$ female flies (n=50 $PEX16^{Ref}$, n=50 $PEX16^{F332del}$, n=50 $PEX16^{R176*}$), along with our *Pex16* null (n=51) and control lines (pink n=48 and purple n=50). (**B**) Lifespan analysis of $PEX16^{Ref\ and\ Variant}$ male flies (n=50 $PEX16^{Ref}$, n=50 $PEX16^{F332del}$, n=48 $PEX16^{R176*}$), along with our *Pex16* null (n=51) and control lines (pink n=50 and purple n=50). (**C**) Bang sensitivity assay at 10 DAE of $PEX16^{Ref\ and\ Variant}$ female flies (n=18 $PEX16^{Ref}$, n=20 $PEX16^{F332del}$, n=17

Disease Models & Mechanisms • Supplementary information

PEX16^{R176*}), along with our *Pex16* null (n=16) and control lines (pink n=20 and purple n=20). (**D**) Bang sensitivity assay at 15 DAE of *PEX16*$^{Ref\ and\ Variant}$ female flies (n=16 *PEX16*^{Ref}, n=18 *PEX16*^{F332del}, n=13 *PEX16*^{R176*}), along with our *Pex16* null (n=12) and control lines (pink n=20 and purple n=20). (**E**) Bang sensitivity assay at 10 DAE of *PEX16*$^{Ref\ and\ Variant}$ male flies (n=17 *PEX16*^{Ref}, n=20 *PEX16*^{F332del}, n=14 *PEX16*^{R176*}), along with our *Pex16* null (n=16) and control lines (pink n=20 and purple n=20). (**F**) Bang sensitivity assay at 15 DAE of *PEX16*$^{Ref\ and\ Variant}$ male flies (n=14 *PEX16*^{Ref}, n=20 *PEX16*^{F332del}, n=10 *PEX16*^{R176*}), along with our *Pex16* null (n=11) and control lines (pink n=20 and purple n=20). (**G**) Climbing assay at 5 DAE of *PEX16*$^{Ref\ and\ Variant}$ female flies (n=20 *PEX16*^{Ref}, n=20 *PEX16*^{F332del}, n=20 *PEX16*^{R176*}), along with our *Pex16* null (n= 20) and control lines (pink n=20 and purple n=20). (**H**) Climbing assay at 10 DAE of *PEX16*$^{Ref\ and\ Variant}$ female flies (n=18 *PEX16*^{Ref}, n=20 *PEX16*^{F332del}, n=17 *PEX16*^{R176*}), along with our *Pex16* null (n=16) and control lines (pink n=20 and purple n=20). (**I**) Climbing assay at 15 DAE of *PEX16*$^{Ref\ and\ Variant}$ female flies (n=16 *PEX16*^{Ref}, n=18 *PEX16*^{F332del}, n=13 *PEX16*^{R176*}), along with our *Pex16* null (n=12) and control lines (pink n=20 and purple n=20). (**J**) Climbing assay at 5 DAE of *PEX16*$^{Ref\ and\ Variant}$ male flies (n=20 *PEX16*^{Ref}, n=20 *PEX16*^{F332del}, n=20 *PEX16*^{R176*}), along with our *Pex16* null (n= 20) and control lines (pink n=20 and purple n=20). (**K**) Climbing assay at 10 DAE of *PEX16*$^{Ref\ and\ Variant}$ male flies (n=17 *PEX16*^{Ref}, n=20 *PEX16*^{F332del}, n=14 *PEX16*^{R176*}), along with our *Pex16* null (n=16) and control lines (pink n=20 and purple n=20). (**L**) Climbing assay at 15 DAE of *PEX16*$^{Ref\ and\ Variant}$ male flies (n=14 *PEX16*^{Ref}, n=20 *PEX16*^{F332del}, n=10 *PEX16*^{R176*}), along with our *Pex16* null (n=11) and control lines (pink n=20 and purple n=20). Ordinary one-way ANOVA with multiple comparisons test
[* = *p*-value is less than 0.05. ** = *p*-value is less than 0.01. *** = *p*-value is less than 0.001. **** = *p*-value is less than 0.0001]

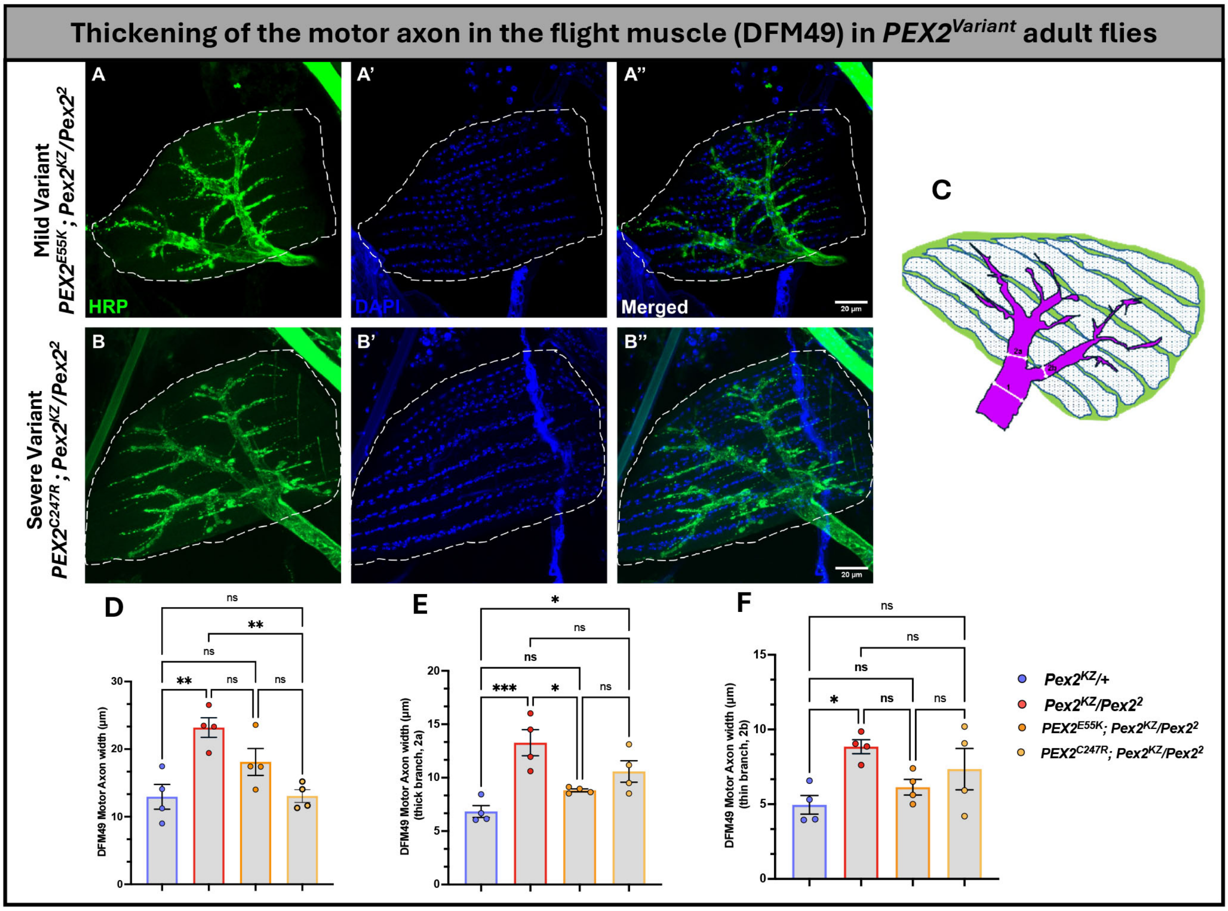

Fig. S3. Thickening of the motor axon in the flight muscle (DFM49) in *PEX2*^{Variant} adult flies.

(**A, A', A'' & A'''**) represents *PEX2* mild variant *PEX2*^{E55K}*; Pex2*^{KZ}*/Pex2*^{2} & (**B, B', B'' & B'''**) represents *PEX2* severe variant *PEX2*^{C247R}*; Pex2*^{KZ}*/Pex2*^{2}. Scale bar corresponds to 20 µm. DFM49 marked with long dashed line. (**C**) Schematic representation of direct flight muscle (DFM49), indicating the motor axon entering the flight muscle (1), thick branch (2a), and thin branch (2b). (**D**) Quantification of the *Pex2* DFM49 motor axon width between four genotypes. (**E**) Quantification of the thick branch of the *Pex2* DFM49 motor axon width between the four genotypes (**F**) Quantification of the thin branch of the *Pex2* DFM49 motor axon width between the four genotypes. n=4 *Pex2*^{KZ}*/+* control, n=4 *Pex2*^{KZ}*/Pex2*^{2}, n=4 *PEX2*^{E55K}, n=4 *PEX2*^{C247R}. Ordinary one-way ANOVA with multiple comparisons test [*$p<0.05$, **$p<0.01$; ***$p<0.001$].

Disease Models & Mechanisms • Supplementary information

ORF	Protected sites	Protected areas	Motifs to avoid
13-1644 [ATG...TAG]	1-8 NotI [GCGGCCGC]<br>1645-1650 XhoI [CTCGAG]		NotI [GCGGCCGC]<br>XhoI [CTCGAG]

```
                   M  A  S  R  K  E  N  A  K  S  A  N  R  V  L  R  I  S  Q
1.    GCGGCCGCCAAAATGGCCAGCCGCAAGGAGAATGCCAAGAGCGCCAATCGCGTGCTGCGCATCAGTCAG
       L  D  A  L  E  L  N  K  A  L  E  Q  L  V  W  S  Q  F  T  Q  C  F  H
70.   CTGGATGCCCTGGAGCTGAACAAGGCCCTGGAGCAGCTCGTGTGGTCCCAGTTCACCCAGTGCTTCCAC
       G  F  K  P  G  L  L  A  R  F  E  P  E  V  K  A  C  L  W  V  F  L  W
139.  GGCTTCAAGCCAGGACTGCTGGCCCGCTTTGAGCCCGAAGTGAAGGCCTGCCTGTGGGTGTTCCTGTGG
       R  F  T  I  Y  S  K  N  A  T  V  G  Q  S  V  L  N  I  K  Y  K  N  D
208.  CGCTTCACCATCTACAGCAAGAACGCCACCGTGGGCCAGAGCGTGCTGAACATCAAGTACAAGAACGAC
       F  S  P  N  L  R  Y  Q  P  P  S  K  N  Q  K  I  W  Y  A  V  C  T  I
277.  TTCAGCCCCAACCTGCGCTACCAGCCCCCGAGCAAGAACCAGAAGATTTGGTACGCCGTGTGCACCATC
       G  G  R  W  L  E  E  R  C  Y  D  L  F  R  N  H  H  L  A  S  F  G  K
346.  GGCGGACGCTGGCTGGAGGAGCGCTGCTACGATCTGTTCCGCAACCACCACCTGGCCAGCTTCGGCAAA
       V  K  Q  C  V  N  F  V  I  G  L  L  K  L  G  G  L  I  N  F  L  I  F
415.  GTGAAGCAGTGCGTGAACTTCGTGATCGGCCTGCTGAAGCTGGGCGGCCTGATCAACTTCCTGATCTTC
       L  Q  R  G  K  F  A  T  L  T  E  R  L  L  G  I  H  S  V  F  C  K  P
484.  CTGCAGCGCGGCAAGTTCGCCACCCTGACCGAGCGCCTGCTGGGCATTCATAGCGTGTTCTGCAAGCCC
       Q  N  I  R  E  V  G  F  E  Y  M  N  R  E  L  L  W  H  G  F  A  E  F
553.  CAGAACATCCGCGAAGTGGGCTTCGAGTACATGAACCGCGAGCTGCTGTGGCACGGCTTCGCCGAGTTT
       L  I  F  L  L  P  L  I  N  V  Q  K  L  K  A  K  L  S  S  W  C  I  P
622.  CTGATTTTCCTGCTGCCGCTGATCAACGTGCAGAAGCTGAAGGCCAAGCTGAGCAGCTGGTGCATCCCA
       L  T  G  A  P  N  S  D  N  T  L  A  T  S  G  K  E  C  A  L  C  G  E
691.  CTGACGGGAGCCCCCAACAGCGATAACACCCTGGCCACCAGCGGAAAGGAGTGCGCCCTGTGCGGAGAG
       W  P  T  M  P  H  T  I  G  C  E  H  I  F  C  Y  F  C  A  K  S  S  F
760.  TGGCCAACCATGCCACACACCATTGGCTGCGAGCACATCTTCTGCTACTTTTGCGCCAAGAGCAGCTTC
       L  F  D  V  Y  F  T  C  P  K  C  G  T  E  V  H  S  L  Q  P  L  K  S
829.  CTGTTCGACGTGTACTTCACGTGCCCCAAGTGCGGCACCGAGGTGCACAGTCTGCAGCCACTGAAGTCC
       G  I  E  M  S  E  V  N  A  L  V  S  K  G  E  E  L  F  T  G  V  V  P
898.  GGCATCGAGATGAGCGAAGTGAACGCCCTGGTGTCCAAGGGCGAGGAGCTGTTTACCGGCGTGGTGCCC
       I  L  V  E  L  D  G  D  V  N  G  H  K  F  S  V  S  G  E  G  E  G  D
967.  ATTCTGGTGGAGCTGGATGGCGACGTGAACGGCCACAAGTTCAGCGTGTCCGGCGAGGGCGAGGGCGAC
       A  T  Y  G  K  L  T  L  K  F  I  C  T  T  G  K  L  P  V  P  W  P  T
1036. GCCACCTATGGAAAGCTGACCCTGAAGTTCATCTGCACCACCGGCAAGCTGCCCGTGCCATGGCCAACC
       L  V  T  T  L  T  Y  G  V  Q  C  F  S  R  Y  P  D  H  M  K  Q  H  D
1105. CTCGTGACCACGCTGACCTATGGCGTGCAGTGCTTCAGCCGCTACCCCGATCACATGAAGCAGCACGAT
       F  F  K  S  A  M  P  E  G  Y  V  Q  E  R  T  I  F  F  K  D  D  G  N
1174. TTCTTCAAGTCCGCCATGCCCGAGGGCTACGTGCAGGAGCGCACCATCTTTTTCAAGGATGACGGCAAC
       Y  K  T  R  A  E  V  K  F  E  G  D  T  L  V  N  R  I  E  L  K  G  I
1243. TACAAGACCCGCGCCGAAGTGAAGTTCGAGGGCGATACCCTCGTGAACCGCATCGAGCTGAAGGGCATC
       D  F  K  E  D  G  N  I  L  G  H  K  L  E  Y  N  Y  N  S  H  N  V  Y
1312. GATTTCAAGGAGGATGGAAACATCCTGGGCCACAAGCTGGAGTACAACTACAACAGCCACAACGTGTAC
       I  M  A  D  K  Q  K  N  G  I  K  V  N  F  K  I  R  H  N  I  E  D  G
1381. ATCATGGCCGACAAGCAGAAGAACGGCATCAAAGTGAACTTCAAGATTCGCCACAACATCGAGGATGGC
       S  V  Q  L  A  D  H  Y  Q  Q  N  T  P  I  G  D  G  P  V  L  L  P  D
1450. AGCGTGCAGCTGGCCGACCACTACCAGCAGAACACCCCCATCGGAGATGGCCCCGTGCTGCTGCCCGAT
       N  H  Y  L  S  T  Q  S  A  L  S  K  D  P  N  E  K  R  D  H  M  V  L
1519. AACCACTACCTGAGTACCCAGAGCGCCCTGAGCAAGGATCCCAACGAGAAGCGCGACCACATGGTGCTG
       L  E  F  V  T  A  A  G  I  T  L  G  M  D  E  L  Y  K  *
1588. CTGGAGTTTGTGACCGCCGCCGGCATTACCCTGGGCATGGATGAGCTGTACAAGTAGCTCGAG
```

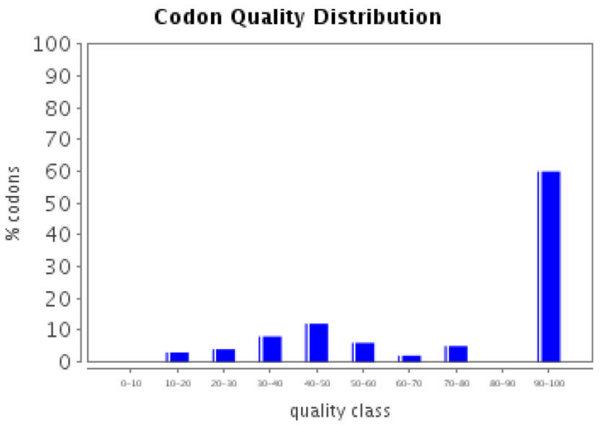

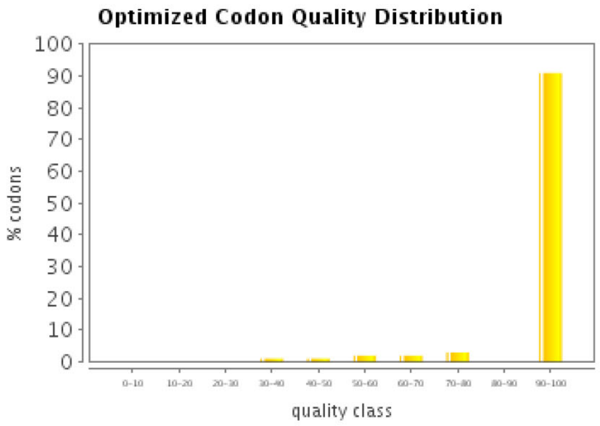

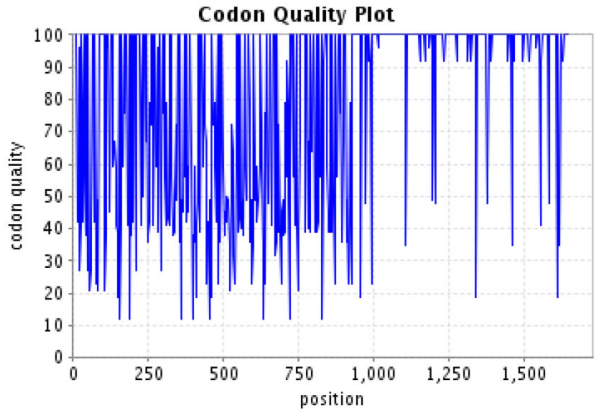

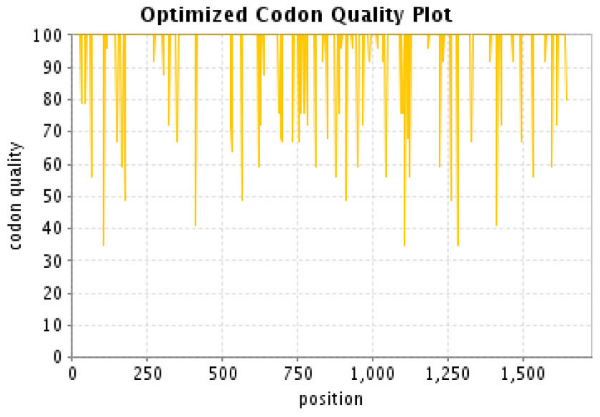

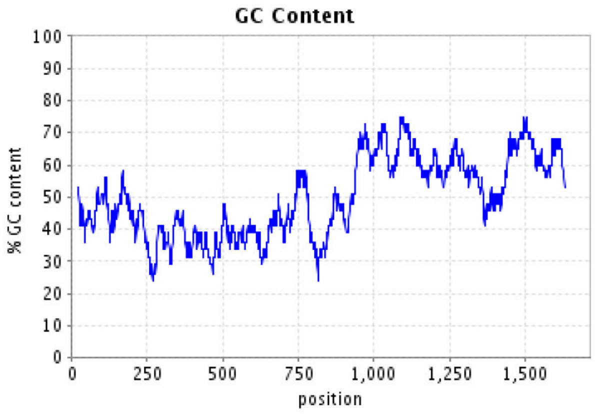

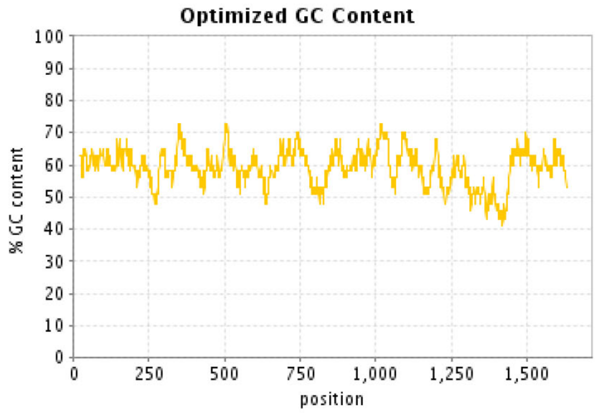

Disease Models & Mechanisms • Supplementary information

ORF	Protected sites	Protected areas	Motifs to avoid
13-1644 [ATG...TAG]	1-8 NotI [GCGGCCGC]<br>1645-1650 XhoI [CTCGAG]		NotI [GCGGCCGC]<br>XhoI [CTCGAG]

```
                   M  A  S  R  K  E  N  A  K  S  A  N  R  V  L  R  I  S  Q
1.    GCGGCCGCCAAAATGGCCAGCCGCAAGGAGAATGCCAAGAGCGCCAATCGCGTGCTGCGCATCAGTCAG
      L  D  A  L  E  L  N  K  A  L  E  Q  L  V  W  S  Q  F  T  Q  C  F  H
70.   CTGGATGCCCTGGAGCTGAACAAGGCCCTGGAGCAGCTCGTGTGGTCCCAGTTCACCCAGTGCTTCCAC
      G  F  K  P  G  L  L  A  R  F  E  P  K  V  K  A  C  L  W  V  F  L  W
139.  GGCTTCAAGCCAGGACTGCTGGCCCGCTTCGAGCCCAAAGTGAAGGCCTGCCTGTGGGTGTTCCTGTGG
      R  F  T  I  Y  S  K  N  A  T  V  G  Q  S  V  L  N  I  K  Y  K  N  D
208.  CGCTTCACCATCTACAGCAAGAACGCCACCGTGGGCCAGAGCGTGCTGAACATCAAGTACAAGAACGAC
      F  S  P  N  L  R  Y  Q  P  P  S  K  N  Q  K  I  W  Y  A  V  C  T  I
277.  TTCAGCCCCAACCTGCGCTACCAGCCCCCGAGCAAGAACCAGAAGATTTGGTACGCCGTGTGCACCATC
      G  G  R  W  L  E  E  R  C  Y  D  L  F  R  N  H  H  L  A  S  F  G  K
346.  GGCGGACGCTGGCTGGAGGAGCGCTGCTACGATCTGTTCCGCAACCACCACCTGGCCAGCTTCGGCAAA
      V  K  Q  C  V  N  F  V  I  G  L  L  K  L  G  G  L  I  N  F  L  I  F
415.  GTGAAGCAGTGCGTGAACTTCGTGATCGGCCTGCTGAAGCTGGGCGGCCTGATCAACTTCCTGATCTTC
      L  Q  R  G  K  F  A  T  L  T  E  R  L  L  G  I  H  S  V  F  C  K  P
484.  CTGCAGCGCGGCAAGTTCGCCACCCTGACCGAGCGCCTGCTGGGCATTCATAGCGTGTTCTGCAAGCCC
      Q  N  I  R  E  V  G  F  E  Y  M  N  R  E  L  L  W  H  G  F  A  E  F
553.  CAGAACATCCGCGAAGTGGGCTTCGAGTACATGAACCGCGAGCTGCTGTGGCACGGCTTCGCCGAGTTT
      L  I  F  L  L  P  L  I  N  V  Q  K  L  K  A  K  L  S  S  W  C  I  P
622.  CTGATTTTCCTGCTGCCGCTGATCAACGTGCAGAAGCTGAAGGCCAAGCTGAGCAGCTGGTGCATCCCA
      L  T  G  A  P  N  S  D  N  T  L  A  T  S  G  K  E  C  A  L  C  G  E
691.  CTGACGGGAGCCCCCAACAGCGATAACACCCTGGCCACCAGCGGAAAGGAGTGCGCCCTGTGCGGAGAG
      W  P  T  M  P  H  T  I  G  C  E  H  I  F  C  Y  F  C  A  K  S  S  F
760.  TGGCCAACCATGCCACACACCATTGGCTGCGAGCACATCTTCTGCTACTTTTGCGCCAAGAGCAGCTTC
      L  F  D  V  Y  F  T  C  P  K  C  G  T  E  V  H  S  L  Q  P  L  K  S
829.  CTGTTCGACGTGTACTTCACGTGCCCCAAGTGCGGCACCGAGGTGCACAGTCTGCAGCCACTGAAGTCC
      G  I  E  M  S  E  V  N  A  L  V  S  K  G  E  E  L  F  T  G  V  V  P
898.  GGCATCGAGATGAGCGAAGTGAACGCCCTGGTGTCCAAGGGCGAGGAGCTGTTTACCGGCGTGGTGCCC
      I  L  V  E  L  D  G  D  V  N  G  H  K  F  S  V  S  G  E  G  E  G  D
967.  ATTCTGGTGGAGCTGGATGGCGACGTGAACGGCCACAAGTTCAGCGTGTCCGGCGAGGGCGAGGGCGAC
      A  T  Y  G  K  L  T  L  K  F  I  C  T  T  G  K  L  P  V  P  W  P  T
1036. GCCACCTATGGAAAGCTGACCCTGAAGTTCATCTGCACCACCGGCAAGCTGCCCGTGCCATGGCCAACC
      L  V  T  T  L  T  Y  G  V  Q  C  F  S  R  Y  P  D  H  M  K  Q  H  D
1105. CTCGTGACCACGCTGACCTATGGCGTGCAGTGCTTCAGCCGCTACCCCGATCACATGAAGCAGCACGAT
      F  F  K  S  A  M  P  E  G  Y  V  Q  E  R  T  I  F  F  K  D  D  G  N
1174. TTCTTCAAGTCCGCCATGCCCGAGGGCTACGTGCAGGAGCGCACCATCTTTTTCAAGGATGACGGCAAC
      Y  K  T  R  A  E  V  K  F  E  G  D  T  L  V  N  R  I  E  L  K  G  I
1243. TACAAGACCCGCGCCGAAGTGAAGTTCGAGGGCGATACCCTCGTGAACCGCATCGAGCTGAAGGGCATC
      D  F  K  E  D  G  N  I  L  G  H  K  L  E  Y  N  Y  N  S  H  N  V  Y
1312. GATTTCAAGGAGGATGGAAACATCCTGGGCCACAAGCTGGAGTACAACTACAACAGCCACAACGTGTAC
      I  M  A  D  K  Q  K  N  G  I  K  V  N  F  K  I  R  H  N  I  E  D  G
1381. ATCATGGCCGACAAGCAGAAGAACGGCATCAAAGTGAACTTCAAGATTCGCCACAACATCGAGGATGGC
      S  V  Q  L  A  D  H  Y  Q  Q  N  T  P  I  G  D  G  P  V  L  L  P  D
1450. AGCGTGCAGCTGGCCGACCACTACCAGCAGAACACCCCCATCGGAGATGGCCCCGTGCTGCTGCCCGAT
      N  H  Y  L  S  T  Q  S  A  L  S  K  D  P  N  E  K  R  D  H  M  V  L
1519. AACCACTACCTGAGTACCCAGAGCGCCCTGAGCAAGGATCCCAACGAGAAGCGCGACCACATGGTGCTG
      L  E  F  V  T  A  A  G  I  T  L  G  M  D  E  L  Y  K  *
1588. CTGGAGTTTGTGACCGCCGCCGGCATTACCCTGGGCATGGATGAGCTGTACAAGTAGCTCGAG
```

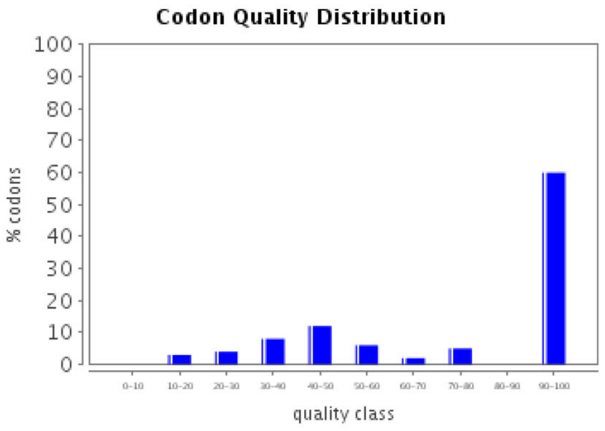

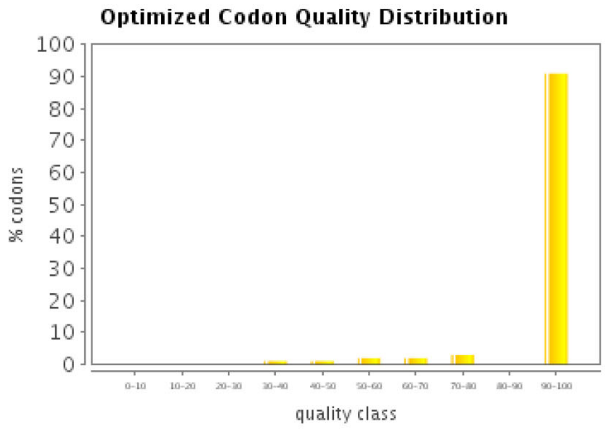

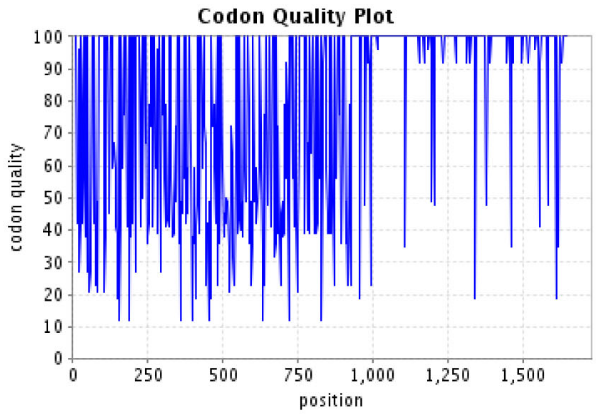

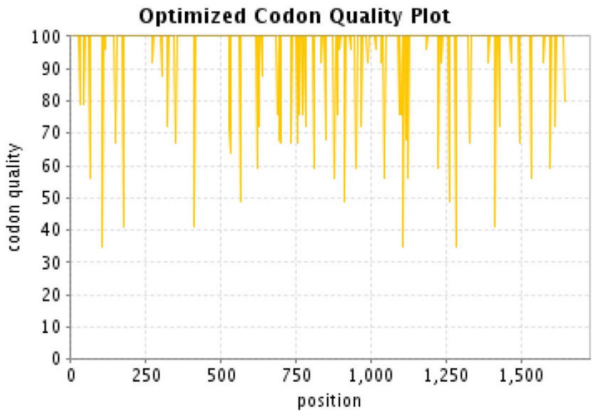

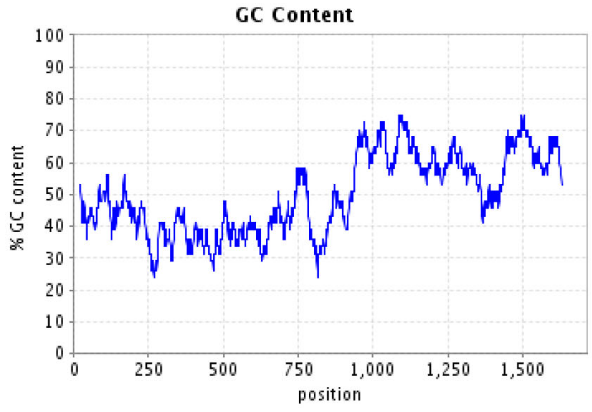

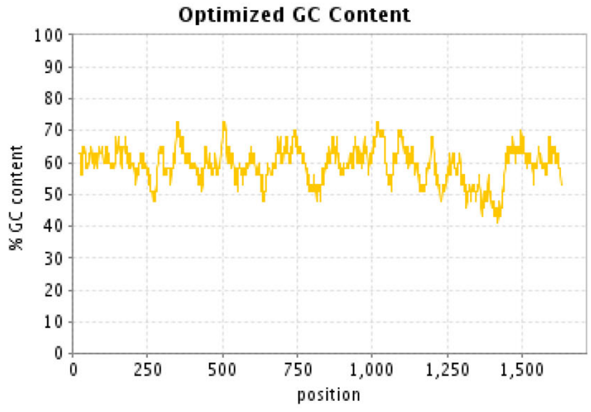

Disease Models & Mechanisms • Supplementary information

ORF	Protected sites	Protected areas	Motifs to avoid
13-1083 [ATG...TAG]	1-8 NotI [GCGGCCGC]<br>1084-1089 XhoI [CTCGAG]		NotI [GCGGCCGC]<br>XhoI [CTCGAG]

```
                  M  A  S  R  K  E  N  A  K  S  A  N  R  V  L  R  I  S  Q
1.    GCGGCCGCCAAAATGGCCAGCCGCAAGGAGAATGCCAAGAGCGCCAATCGCGTGCTGCGCATCAGTCAG
      L  D  A  L  E  L  N  K  A  L  E  Q  L  V  W  S  Q  F  T  Q  C  F  H
70.   CTGGATGCCCTGGAGCTGAACAAGGCCCTGGAGCAGCTCGTGTGGTCCCAGTTCACCCAGTGCTTCCAC
      G  F  K  P  G  L  L  A  R  F  E  P  E  V  K  A  C  L  W  V  F  L  W
139.  GGCTTCAAGCCAGGACTGCTGGCCCGCTTTGAGCCCGAAGTGAAGGCCTGCCTGTGGGTGTTCCTGTGG
      R  F  T  I  Y  S  K  N  A  T  V  G  Q  S  V  L  N  I  K  Y  K  N  D
208.  CGCTTCACCATCTACAGCAAGAACGCCACCGTGGGCCAGAGCGTGCTGAACATCAAGTACAAGAACGAC
      F  S  P  N  L  R  Y  Q  P  P  S  K  N  Q  K  I  W  Y  A  V  C  T  I
277.  TTCAGCCCCAACCTGCGCTACCAGCCCCCGAGCAAGAACCAGAAGATTTGGTACGCCGTGTGCACCATC
      G  G  R  W  L  E  E  V  S  K  G  E  E  L  F  T  G  V  V  P  I  L  V
346.  GGCGGACGCTGGCTGGAGGAGGTGTCCAAGGGCGAGGAGCTGTTTACCGGCGTGGTGCCCATTCTGGTG
      E  L  D  G  D  V  N  G  H  K  F  S  V  S  G  E  G  E  G  D  A  T  Y
415.  GAGCTGGATGGCGACGTGAACGGCCACAAGTTCAGCGTGTCCGGCGAGGGCGAGGGCGACGCCACCTAT
      G  K  L  T  L  K  F  I  C  T  T  G  K  L  P  V  P  W  P  T  L  V  T
484.  GGAAAGCTGACCCTGAAGTTCATCTGCACCACCGGCAAGCTGCCCGTGCCATGGCCAACCCTCGTGACC
      T  L  T  Y  G  V  Q  C  F  S  R  Y  P  D  H  M  K  Q  H  D  F  F  K
553.  ACCCTGACCTATGGCGTGCAGTGCTTCAGCCGCTACCCCGATCACATGAAGCAGCACGATTTCTTCAAG
      S  A  M  P  E  G  Y  V  Q  E  R  T  I  F  F  K  D  D  G  N  Y  K  T
622.  TCCGCCATGCCCGAGGGCTACGTGCAGGAGCGCACCATCTTTTTCAAGGATGACGGCAACTACAAGACC
      R  A  E  V  K  F  E  G  D  T  L  V  N  R  I  E  L  K  G  I  D  F  K
691.  CGCGCCGAAGTGAAGTTCGAGGGCGATACCCTCGTGAACCGCATCGAGCTGAAGGGCATCGATTTCAAG
      E  D  G  N  I  L  G  H  K  L  E  Y  N  Y  N  S  H  N  V  Y  I  M  A
760.  GAGGATGGAAACATCCTGGGCCACAAGCTGGAGTACAACTACAACAGCCACAACGTGTACATCATGGCC
      D  K  Q  K  N  G  I  K  V  N  F  K  I  R  H  N  I  E  D  G  S  V  Q
829.  GACAAGCAGAAGAACGGCATCAAAGTGAACTTCAAGATTCGCCACAACATCGAGGATGGCAGCGTGCAG
      L  A  D  H  Y  Q  Q  N  T  P  I  G  D  G  P  V  L  L  P  D  N  H  Y
898.  CTGGCCGATCACTACCAGCAGAACACCCCAATCGGCGACGGCCCAGTGCTGCTGCCCGATAACCATTAC
      L  S  T  Q  S  A  L  S  K  D  P  N  E  K  R  D  H  M  V  L  L  E  F
967.  CTGAGCACCCAGAGCGCCCTGAGCAAGGATCCCAACGAGAAGCGCGACCACATGGTGCTGCTGGAGTTT
      V  T  A  A  G  I  T  L  G  M  D  E  L  Y  K  *
1036. GTGACCGCCGCCGGCATTACCCTGGGCATGGATGAGCTGTACAAGTAGCTCGAG
```

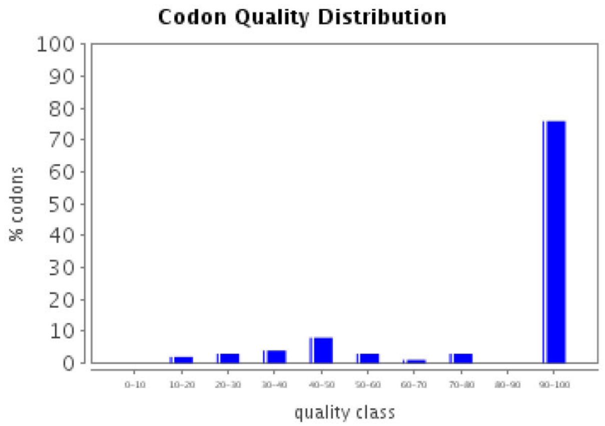

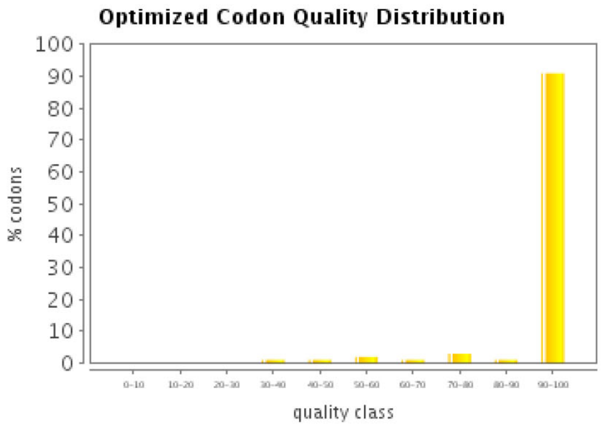

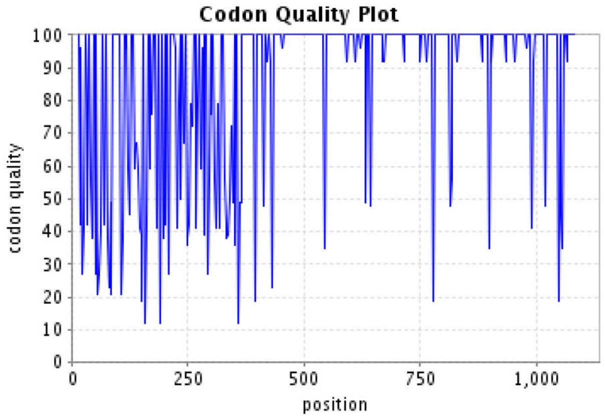

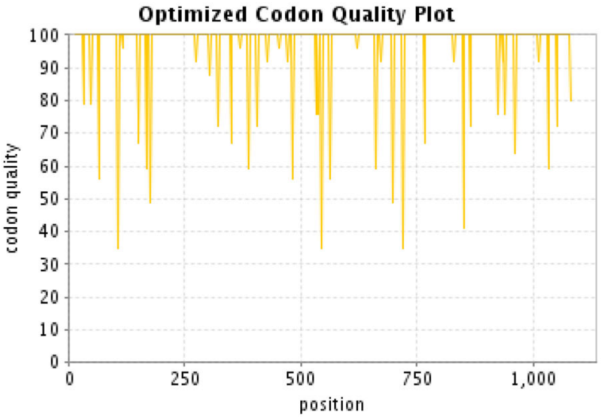

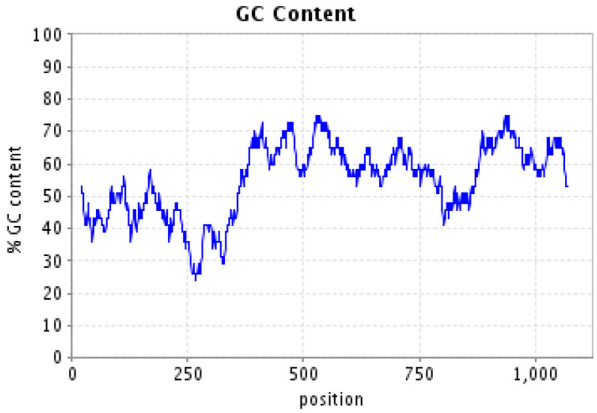

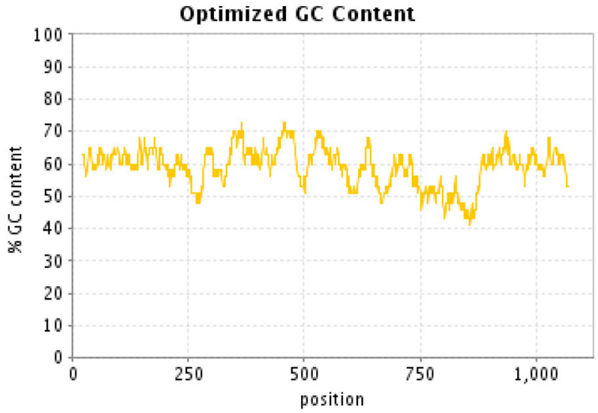

Disease Models & Mechanisms • Supplementary information

ORF	Protected sites	Protected areas	Motifs to avoid
13-1395 [ATG...TAG]	1-8 NotI [GCGGCCGC]<br>1396-1401 XhoI [CTCGAG]		NotI [GCGGCCGC]<br>XhoI [CTCGAG]

```
                   M  A  S  R  K  E  N  A  K  S  A  N  R  V  L  R  I  S  Q
1.     GCGGCCGCCAAAATGGCCAGCCGCAAGGAGAATGCCAAGAGCGCCAATCGCGTGCTGCGCATCAGTCAG
       L  D  A  L  E  L  N  K  A  L  E  Q  L  V  W  S  Q  F  T  Q  C  F  H
70.    CTGGATGCCCTGGAGCTGAACAAGGCCCTGGAGCAGCTCGTGTGGTCCCAGTTCACCCAGTGCTTCCAC
       G  F  K  P  G  L  L  A  R  F  E  P  E  V  K  A  C  L  W  V  F  L  W
139.   GGCTTCAAGCCAGGACTGCTGGCCCGCTTTGAGCCCGAAGTGAAGGCCTGCCTGTGGGTGTTCCTGTGG
       R  F  T  I  Y  S  K  N  A  T  V  G  Q  S  V  L  N  I  K  Y  K  N  D
208.   CGCTTCACCATCTACAGCAAGAACGCCACCGTGGGCCAGAGCGTGCTGAACATCAAGTACAAGAACGAC
       F  S  P  N  L  R  Y  Q  P  P  S  K  N  Q  K  I  W  Y  A  V  C  T  I
277.   TTCAGCCCCAACCTGCGCTACCAGCCCCCGAGCAAGAACCAGAAGATTTGGTACGCCGTGTGCACCATC
       G  G  R  W  L  E  E  R  C  Y  D  L  F  R  N  H  H  L  A  S  F  G  K
346.   GGCGGACGCTGGCTGGAGGAGCGCTGCTACGATCTGTTCCGCAACCACCACCTGGCCAGCTTCGGCAAA
       V  K  Q  C  V  N  F  V  I  G  L  L  K  L  G  G  L  I  N  F  L  I  F
415.   GTGAAGCAGTGCGTGAACTTCGTGATCGGCCTGCTGAAGCTGGGCGGCCTGATCAACTTCCTGATCTTC
       L  Q  R  G  K  F  A  T  L  T  E  R  L  L  G  I  H  S  V  F  C  K  P
484.   CTGCAGCGCGGCAAGTTCGCCACCCTGACCGAGCGCCTGCTGGGCATTCATAGCGTGTTCTGCAAGCCC
       Q  N  I  R  E  V  G  F  E  Y  M  N  R  E  L  L  W  H  G  F  A  E  F
553.   CAGAACATCCGCGAAGTGGGCTTCGAGTACATGAACCGCGAGCTGCTGTGGCACGGCTTCGCCGAGTTT
       L  I  F  L  L  P  L  I  N  V  Q  K  L  K  A  K  L  S  S  V  S  K  G
622.   CTGATTTTCCTGCTGCCGCTGATCAACGTGCAGAAGCTGAAGGCCAAGCTGAGCAGCGTGTCCAAGGGC
       E  E  L  F  T  G  V  V  P  I  L  V  E  L  D  G  D  V  N  G  H  K  F
691.   GAGGAGCTGTTTACCGGCGTGGTGCCCATTCTGGTGGAGCTGGATGGCGACGTGAACGGCCACAAGTTC
       S  V  S  G  E  G  E  G  D  A  T  Y  G  K  L  T  L  K  F  I  C  T  T
760.   TCCGTGTCCGGCGAGGGCGAGGGCGACGCCACCTATGGAAAGCTGACCCTGAAGTTCATCTGCACCACC
       G  K  L  P  V  P  W  P  T  L  V  T  T  L  T  Y  G  V  Q  C  F  S  R
829.   GGCAAGCTGCCCGTGCCATGGCCAACCCTCGTGACCACGCTGACCTATGGCGTGCAGTGCTTCAGCCGC
       Y  P  D  H  M  K  Q  H  D  F  F  K  S  A  M  P  E  G  Y  V  Q  E  R
898.   TACCCCGATCACATGAAGCAGCACGATTTCTTCAAGTCCGCCATGCCCGAGGGCTACGTGCAGGAGCGC
       T  I  F  F  K  D  D  G  N  Y  K  T  R  A  E  V  K  F  E  G  D  T  L
967.   ACCATCTTTTTCAAGGATGACGGCAACTACAAGACCCGCGCCGAAGTGAAGTTCGAGGGCGATACCCTC
       V  N  R  I  E  L  K  G  I  D  F  K  E  D  G  N  I  L  G  H  K  L  E
1036.  GTGAACCGCATCGAGCTGAAGGGCATCGATTTCAAGGAGGATGGAAACATCCTGGGCCACAAGCTGGAG
       Y  N  Y  N  S  H  N  V  Y  I  M  A  D  K  Q  K  N  G  I  K  V  N  F
1105.  TACAACTACAACAGCCACAACGTGTACATCATGGCCGACAAGCAGAAGAACGGCATCAAAGTGAACTTC
       K  I  R  H  N  I  E  D  G  S  V  Q  L  A  D  H  Y  Q  Q  N  T  P  I
1174.  AAGATTCGCCACAACATCGAGGATGGCAGCGTGCAGCTGGCCGACCACTACCAGCAGAACACCCCCATC
       G  D  G  P  V  L  L  P  D  N  H  Y  L  S  T  Q  S  A  L  S  K  D  P
1243.  GGAGATGGCCCCGTGCTGCTGCCCGATAACCACTACCTGAGTACCCAGAGCGCCCTGAGCAAGGATCCC
       N  E  K  R  D  H  M  V  L  L  E  F  V  T  A  A  G  I  T  L  G  M  D
1312.  AACGAGAAGCGCGACCACATGGTGCTGCTGGAGTTTGTGACCGCCGCCGGCATTACCCTGGGCATGGAT
       E  L  Y  K  *
1381.  GAGCTGTACAAGTAGCTCGAG
```

Disease Models & Mechanisms • Supplementary information

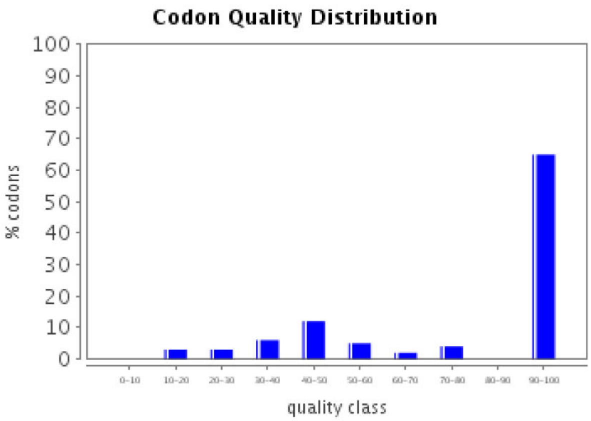

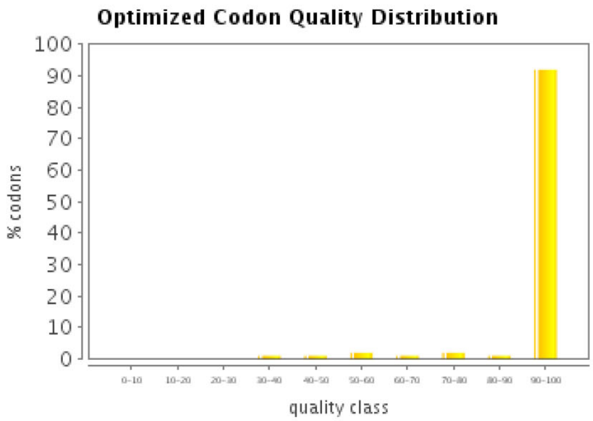

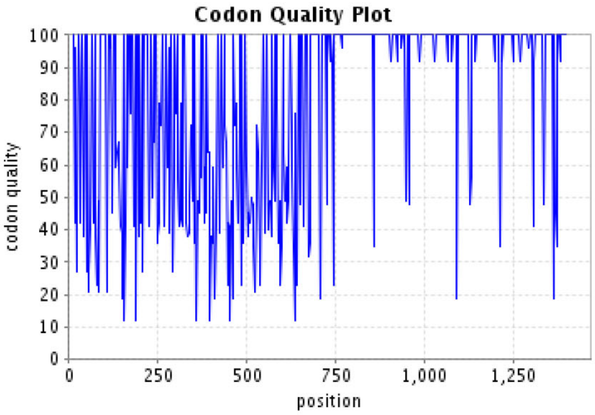

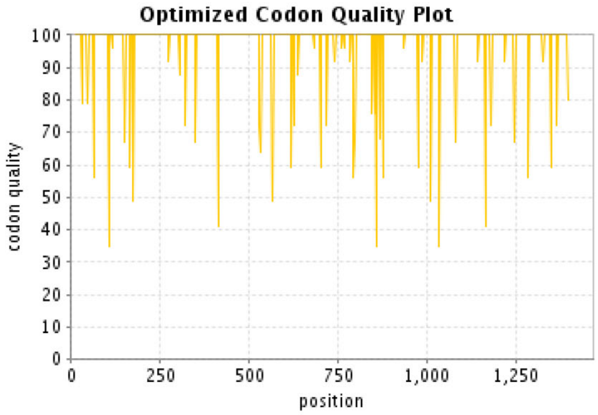

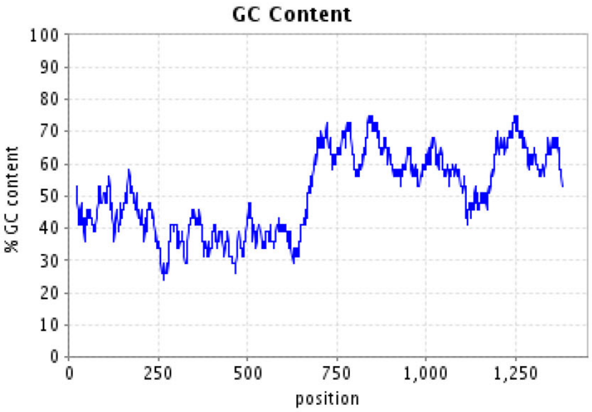

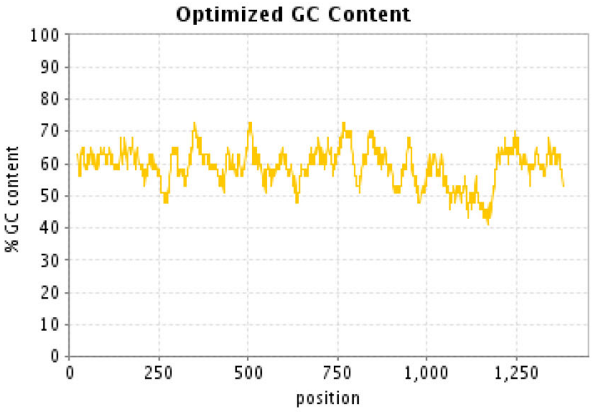

Disease Models & Mechanisms • Supplementary information

ORF	Protected sites	Protected areas	Motifs to avoid
13-1644 [ATG...TAG]	1-8 NotI [GCGGCCGC]<br>1645-1650 XhoI [CTCGAG]		NotI [GCGGCCGC]<br>XhoI [CTCGAG]

```
            M A S R K E N A K S A N R V L R I S Q
1.    GCGGCCGCCAAAATGGCCAGCCGCAAGGAGAATGCCAAGAGCGCCAATCGCGTGCTGCGCATCAGTCAG
      L D A L E L N K A L E Q L V W S Q F T Q C F H
70.   CTGGATGCCCTGGAGCTGAACAAGGCCCTGGAGCAGCTCGTGTGGTCCCAGTTCACCCAGTGCTTCCAC
      G F K P G L L A R F E P E V K A C L W V F L W
139.  GGCTTCAAGCCAGGACTGCTGGCCCGCTTTGAGCCCGAAGTGAAGGCCTGCCTGTGGGTGTTCCTGTGG
      R F T I Y S K N A T V G Q S V L N I K Y K N D
208.  CGCTTCACCATCTACAGCAAGAACGCCACCGTGGGCCAGAGCGTGCTGAACATCAAGTACAAGAACGAC
      F S P N L R Y Q P P S K N Q K I W Y A V C T I
277.  TTCAGCCCCAACCTGCGCTACCAGCCCCCGAGCAAGAACCAGAAGATTTGGTACGCCGTGTGCACCATC
      G G R W L E E R C Y D L F R N H H L A S F G K
346.  GGCGGACGCTGGCTGGAGGAGCGCTGCTACGATCTGTTCCGCAACCACCACCTGGCCAGCTTCGGCAAA
      V K Q C V N F V I G L L K L G G L I N F L I F
415.  GTGAAGCAGTGCGTGAACTTCGTGATCGGCCTGCTGAAGCTGGGCGGCCTGATCAACTTCCTGATCTTC
      L Q R G K F A T L T E R L L G I H S V F C K P
484.  CTGCAGCGCGGCAAGTTCGCCACCCTGACCGAGCGCCTGCTGGGCATTCATAGCGTGTTCTGCAAGCCC
      Q N I R E V G F E Y M N R E L L W H G F A E F
553.  CAGAACATCCGCGAAGTGGGCTTCGAGTACATGAACCGCGAGCTGCTGTGGCACGGCTTCGCCGAGTTT
      L I F L L P L I N V Q K L K A K L S S W C I P
622.  CTGATTTTCCTGCTGCCGCTGATCAACGTGCAGAAGCTGAAGGCCAAGCTGAGCAGCTGGTGCATCCCA
      L T G A P N S D N T L A T S G K E C A L R G E
691.  CTGACGGGAGCCCCCAACAGCGATAACACCCTGGCCACCAGCGGAAAGGAGTGCGCCCTGCGCGGAGAG
      W P T M P H T I G C E H I F C Y F C A K S S F
760.  TGGCCAACCATGCCACACACCATTGGCTGCGAGCACATCTTCTGCTACTTTTGCGCCAAGAGCAGCTTC
      L F D V Y F T C P K C G T E V H S L Q P L K S
829.  CTGTTCGACGTGTACTTCACGTGCCCCAAGTGCGGCACCGAGGTGCACAGTCTGCAGCCACTGAAGTCC
      G I E M S E V N A L V S K G E E L F T G V V P
898.  GGCATCGAGATGAGCGAAGTGAACGCCCTGGTGTCCAAGGGCGAGGAGCTGTTTACCGGCGTGGTGCCC
      I L V E L D G D V N G H K F S V S G E G E G D
967.  ATTCTGGTGGAGCTGGATGGCGACGTGAACGGCCACAAGTTCAGCGTGTCCGGCGAGGGCGAGGGCGAC
      A T Y G K L T L K F I C T T G K L P V P W P T
1036. GCCACCTATGGAAAGCTGACCCTGAAGTTCATCTGCACCACCGGCAAGCTGCCCGTGCCATGGCCAACC
      L V T T L T Y G V Q C F S R Y P D H M K Q H D
1105. CTCGTGACCACGCTGACCTATGGCGTGCAGTGCTTCAGCCGCTACCCCGATCACATGAAGCAGCACGAT
      F F K S A M P E G Y V Q E R T I F F K D D G N
1174. TTCTTCAAGTCCGCCATGCCCGAGGGCTACGTGCAGGAGCGCACCATCTTTTTCAAGGATGACGGCAAC
      Y K T R A E V K F E G D T L V N R I E L K G I
1243. TACAAGACCCGCGCCGAAGTGAAGTTCGAGGGCGATACCCTCGTGAACCGCATCGAGCTGAAGGGCATC
      D F K E D G N I L G H K L E Y N Y N S H N V Y
1312. GATTTCAAGGAGGATGGAAACATCCTGGGCCACAAGCTGGAGTACAACTACAACAGCCACAACGTGTAC
      I M A D K Q K N G I K V N F K I R H N I E D G
1381. ATCATGGCCGACAAGCAGAAGAACGGCATCAAAGTGAACTTCAAGATTCGCCACAACATCGAGGATGGC
      S V Q L A D H Y Q Q N T P I G D G P V L L P D
1450. AGCGTGCAGCTGGCCGACCACTACCAGCAGAACACCCCCATCGGAGATGGCCCCGTGCTGCTGCCCGAT
      N H Y L S T Q S A L S K D P N E K R D H M V L
1519. AACCACTACCTGAGTACCCAGAGCGCCCTGAGCAAGGATCCCAACGAGAAGCGCGACCACATGGTGCTG
      L E F V T A A G I T L G M D E L Y K *
1588. CTGGAGTTTGTGACCGCCGCCGGCATTACCCTGGGCATGGATGAGCTGTACAAGTAGCTCGAG
```

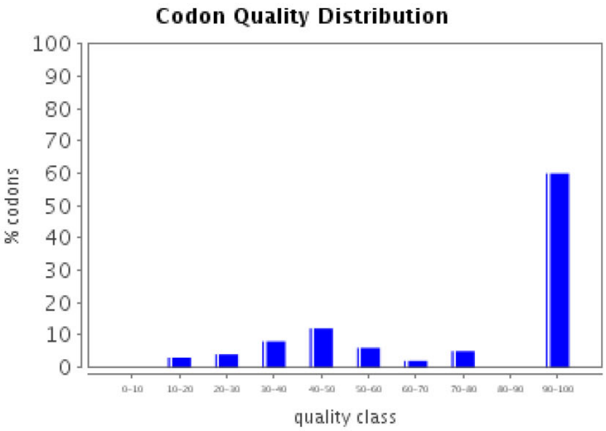
Codon Quality Distribution
% codons
quality class
0-10
10-20
20-30
30-40
40-50
50-60
60-70
70-80
80-90
90-100

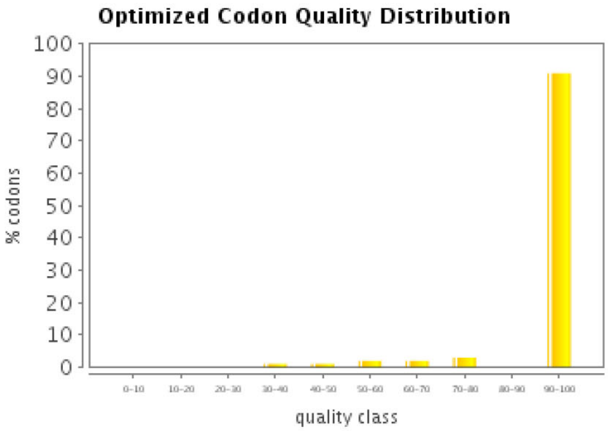
Optimized Codon Quality Distribution
% codons
quality class
0-10
10-20
20-30
30-40
40-50
50-60
60-70
70-80
80-90
90-100

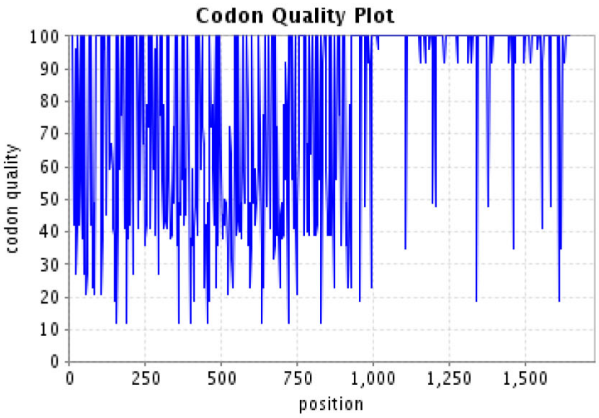
Codon Quality Plot
codon quality
position

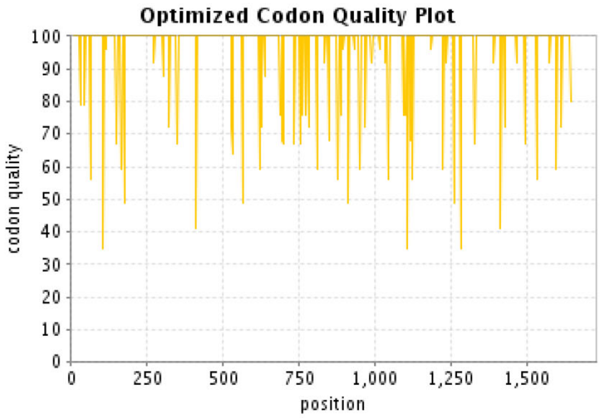
Optimized Codon Quality Plot
codon quality
position

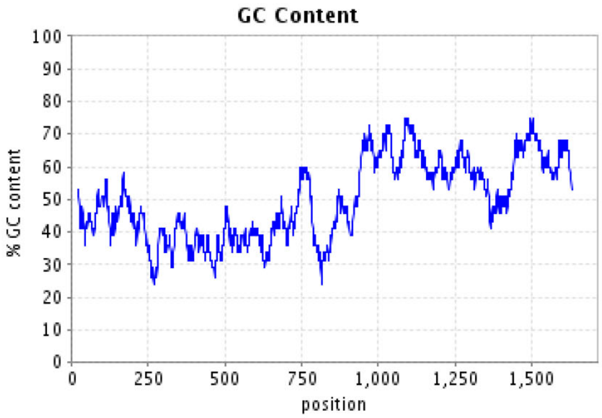
GC Content
% GC content
position

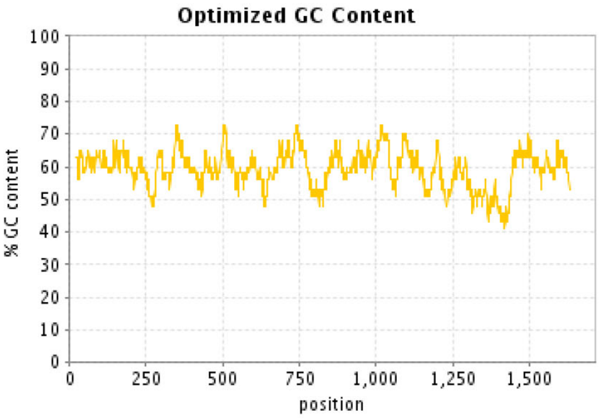
Optimized GC Content
% GC content
position

ORF	Protected sites	Protected areas	Motifs to avoid
13-1065 [ATG...TAG]	1-8 NotI [GCGGCCGC]<br>1066-1071 XhoI [CTCGAG]		NotI [GCGGCCGC]<br>XhoI [CTCGAG]

```
            M  G  K  P  I  P  N  P  L  L  G  L  D  S  T  E  K  L  R
1.    GCGGCCGCCAAAATGGGCAAGCCCATCCCCAATCCACTGCTGGGCCTGGATAGCACCGAGAAGCTGCGC
      L  L  G  L  R  Y  Q  E  Y  V  T  R  H  P  A  A  T  A  Q  L  E  T  A
70.   CTGCTGGGACTGCGCTACCAGGAGTATGTGACCCGCCATCCAGCCGCCACCGCCCAGCTGGAGACCGCC
      V  R  G  F  S  Y  L  L  A  G  R  F  A  D  S  H  E  L  S  E  L  V  Y
139.  GTGCGCGGATTTTCCTATCTGCTGGCCGGACGCTTCGCCGATAGCCACGAGCTGAGCGAGCTGGTGTAC
      S  A  S  N  L  L  V  L  L  N  D  G  I  L  R  K  E  L  R  K  K  L  P
208.  AGCGCCAGCAACCTGCTGGTGCTGCTGAACGATGGCATCCTGCGCAAGGAGCTGCGCAAGAAGCTGCCC
      V  S  L  S  Q  Q  K  L  L  T  W  L  S  V  L  E  C  V  E  V  F  M  E
277.  GTGTCCCTGAGCCAGCAGAAGCTGCTGACCTGGCTGTCCGTGCTGGAGTGCGTGGAGGTGTTCATGGAG
      M  G  A  A  K  V  W  G  E  V  G  R  W  L  V  I  A  L  I  Q  L  A  K
346.  ATGGGAGCCGCCAAAGTGTGGGGCGAAGTGGGACGCTGGCTCGTGATCGCCCTGATCCAGCTGGCCAAG
      A  V  L  R  M  L  L  L  L  W  F  K  A  G  L  Q  T  S  P  P  I  V  P
415.  GCCGTGCTGCGCATGTTGCTGCTGCTGTGGTTCAAGGCCGGACTGCAGACCAGCCCCCCAATCGTGCCA
      L  D  R  E  T  Q  A  Q  P  P  D  G  D  H  S  P  G  N  H  E  Q  S  Y
484.  CTGGATCGCGAGACGCAGGCCCAGCCACCAGATGGCGATCACTCCCCAGGCAACCACGAGCAGAGCTAC
      V  G  K  R  S  N  R  V  V  R  T  L  Q  N  T  P  S  L  H  S  R  H  W
553.  GTGGGCAAGCGCTCCAATCGCGTCGTGCGCACCCTGCAGAATACCCCCAGTCTGCACAGCCGCCATTGG
      G  A  P  Q  Q  R  E  G  R  Q  Q  Q  H  H  E  E  L  S  A  T  P  T  P
622.  GGAGCCCCCCAGCAGCGCGAGGGACGCCAGCAGCAGCATCACGAGGAGCTGAGTGCCACCCCAACCCCA
      L  G  L  Q  E  T  I  A  E  F  L  Y  I  A  R  P  L  L  H  L  L  S  L
691.  CTGGGACTGCAGGAGACGATCGCCGAGTTCCTGTACATTGCCCGCCCACTGCTGCATCTGCTGAGCCTG
      G  L  W  G  Q  R  S  W  K  P  W  L  L  A  G  V  V  D  V  T  S  L  S
760.  GGACTGTGGGGACAGCGCAGTTGGAAGCCCTGGCTGCTGGCCGGCGTGGTGGATGTGACCAGTCTGAGC
      L  L  S  D  R  K  G  L  T  R  R  E  R  R  E  L  R  R  R  T  I  L  L
829.  CTGCTGAGCGATCGCAAGGGACTGACCCGCCGCGAGCGCCGCGAGCTGCGCCGCCGCACCATTCTGCTG
      L  Y  Y  L  L  R  S  P  F  Y  D  R  F  S  E  A  R  I  L  F  L  L  Q
898.  CTGTACTATCTGCTGCGCTCCCCCTTCTACGATCGCTTCTCCGAGGCCCGCATCCTGTTTCTGCTGCAG
      L  L  A  D  H  V  P  G  V  G  L  V  T  R  P  L  M  D  Y  L  P  T  W
967.  CTGCTGGCCGATCACGTGCCCGGCGTGGGACTCGTGACGCGCCCACTGATGGATTACCTGCCCACCTGG
      Q  K  I  Y  F  Y  S  W  G  *
1036. CAGAAGATCTACTTCTACAGCTGGGGCTAGCTCGAG
```

Disease Models & Mechanisms • Supplementary information

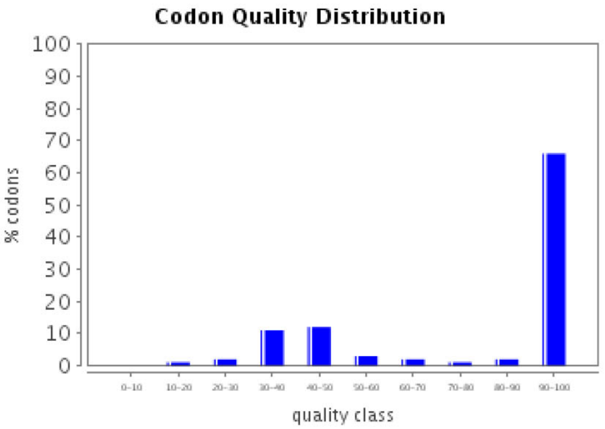

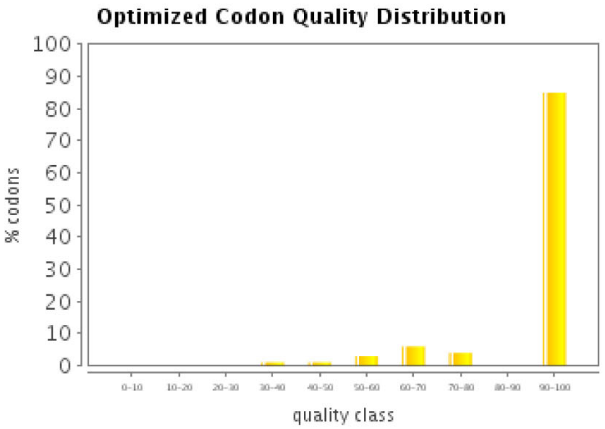

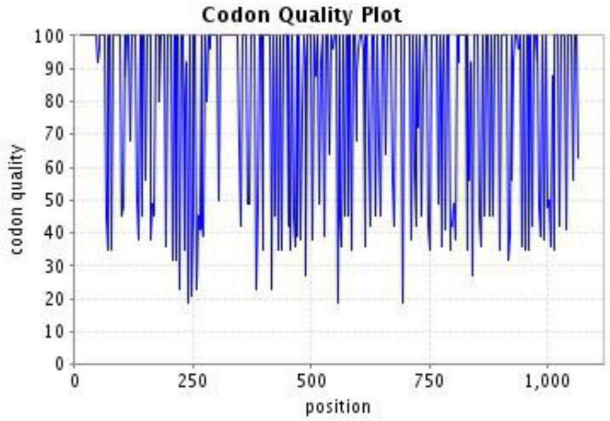

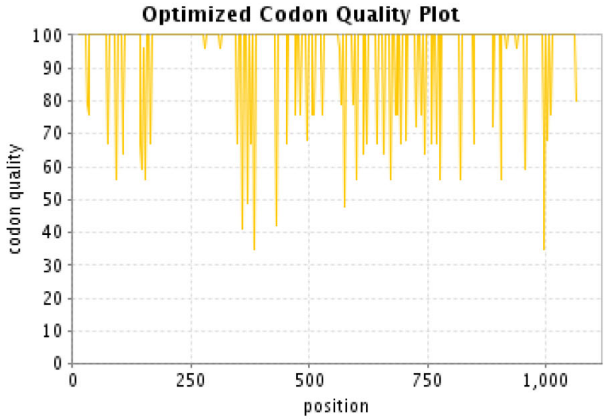

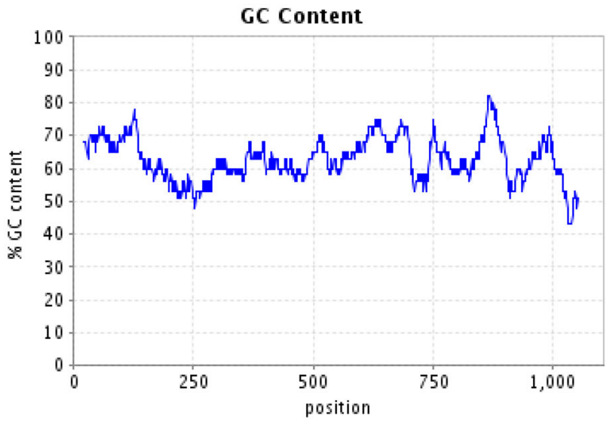

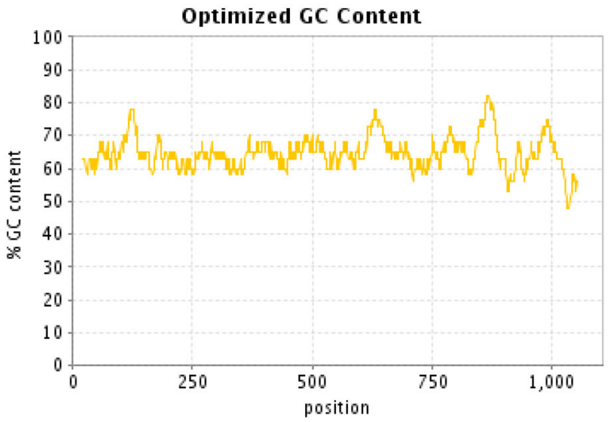

Disease Models & Mechanisms • Supplementary information

ORF	Protected sites	Protected areas	Motifs to avoid
13-582 [ATG...TAA]	1-8 NotI [GCGGCCGC]<br>583-588 XhoI [CTCGAG]		NotI [GCGGCCGC]<br>XhoI [CTCGAG]

```
                M  G  K  P  I  P  N  P  L  L  G  L  D  S  T  E  K  L  R
  1.   GCGGCCGCCAAAATGGGCAAGCCCATCCCCAATCCACTGCTGGGCCTGGATAGCACCGAGAAGCTGCGC
        L  L  G  L  R  Y  Q  E  Y  V  T  R  H  P  A  A  T  A  Q  L  E  T  A
 70.   CTGCTGGGACTGCGCTACCAGGAGTATGTGACCCGCCATCCAGCCGCCACCGCCCAGCTGGAGACCGCC
        V  R  G  F  S  Y  L  L  A  G  R  F  A  D  S  H  E  L  S  E  L  V  Y
139.   GTGCGCGGATTTTCCTATCTGCTGGCCGGACGCTTCGCCGATAGCCACGAGCTGAGCGAGCTGGTGTAC
        S  A  S  N  L  L  V  L  L  N  D  G  I  L  R  K  E  L  R  K  K  L  P
208.   AGCGCCAGCAACCTGCTGGTGCTGCTGAACGATGGCATCCTGCGCAAGGAGCTGCGCAAGAAGCTGCCC
        V  S  L  S  Q  Q  K  L  L  T  W  L  S  V  L  E  C  V  E  V  F  M  E
277.   GTGTCCCTGAGCCAGCAGAAGCTGCTGACCTGGCTGTCCGTGCTGGAGTGCGTGGAGGTGTTCATGGAG
        M  G  A  A  K  V  W  G  E  V  G  R  W  L  V  I  A  L  I  Q  L  A  K
346.   ATGGGAGCCGCCAAAGTGTGGGGCGAAGTGGGACGCTGGCTCGTGATCGCCCTGATCCAGCTGGCCAAG
        A  V  L  R  M  L  L  L  L  W  F  K  A  G  L  Q  T  S  P  P  I  V  P
415.   GCCGTGCTGCGCATGTTGCTGCTGCTGTGGTTCAAGGCCGGACTGCAGACCAGCCCCCCAATCGTGCCA
        L  D  R  E  T  Q  A  Q  P  P  D  G  D  H  S  P  G  N  H  E  Q  S  Y
484.   CTGGATCGCGAGACGCAGGCCCAGCCACCAGATGGCGATCACTCCCCAGGCAACCACGAGCAGAGCTAC
        V  G  K  R  S  N  R  V  V  *
553.   GTGGGCAAGCGCTCCAACCGCGTGGTGTAACTCGAG
```

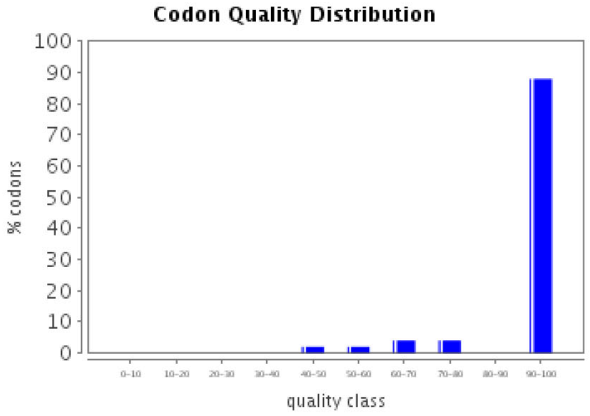

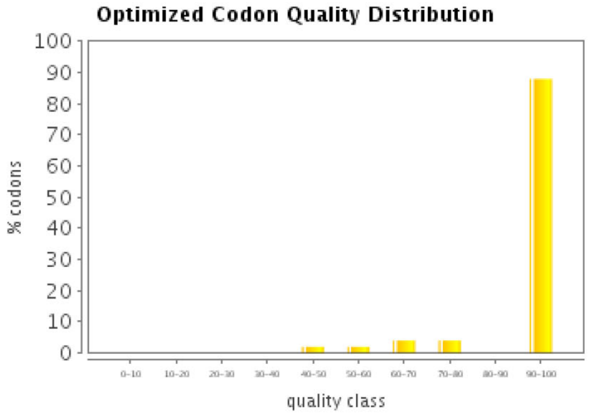

Disease Models & Mechanisms • Supplementary information

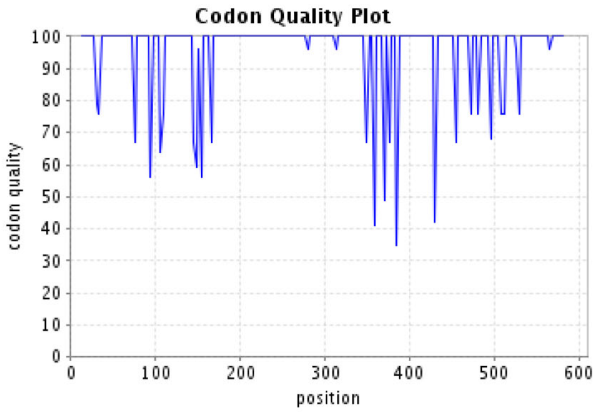

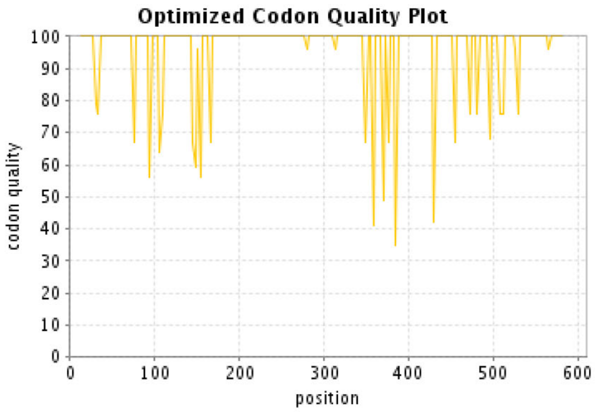

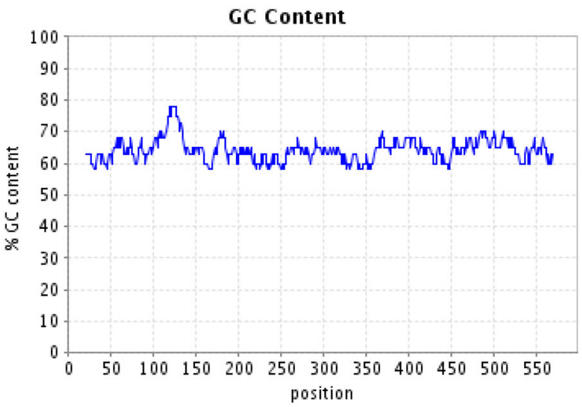

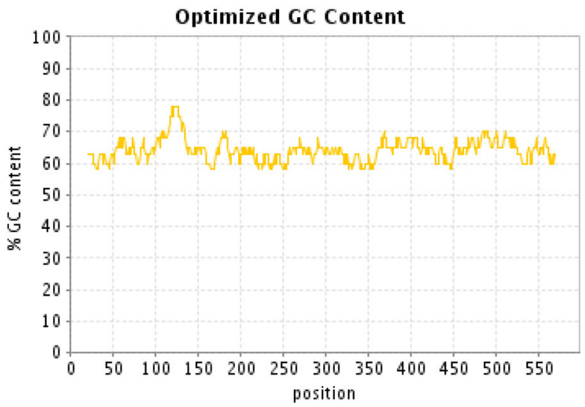

Disease Models & Mechanisms • Supplementary information

ORF	Protected sites	Protected areas	Motifs to avoid
13-1062 [ATG...TAA]	1-8 NotI [GCGGCCGC]<br>1063-1068 XhoI [CTCGAG]		NotI [GCGGCCGC]<br>XhoI [CTCGAG]

```
                    M  G  K  P  I  P  N  P  L  L  G  L  D  S  T  E  K  L  R
   1. GCGGCCGCCAAAATGGGCAAGCCCATCCCCAATCCACTGCTGGGCCTGGATAGCACCGAGAAGCTGCGC
      L  L  G  L  R  Y  Q  E  Y  V  T  R  H  P  A  A  T  A  Q  L  E  T  A
  70. CTGCTGGGACTGCGCTACCAGGAGTATGTGACCCGCCATCCAGCCGCCACCGCCCAGCTGGAGACCGCC
      V  R  G  F  S  Y  L  L  A  G  R  F  A  D  S  H  E  L  S  E  L  V  Y
 139. GTGCGCGGATTTTCCTATCTGCTGGCCGGACGCTTCGCCGATAGCCACGAGCTGAGCGAGCTGGTGTAC
      S  A  S  N  L  L  V  L  L  N  D  G  I  L  R  K  E  L  R  K  K  L  P
 208. AGCGCCAGCAACCTGCTGGTGCTGCTGAACGATGGCATCCTGCGCAAGGAGCTGCGCAAGAAGCTGCCC
      V  S  L  S  Q  Q  K  L  L  T  W  L  S  V  L  E  C  V  E  V  F  M  E
 277. GTGTCCCTGAGCCAGCAGAAGCTGCTGACCTGGCTGTCCGTGCTGGAGTGCGTGGAGGTGTTCATGGAG
      M  G  A  A  K  V  W  G  E  V  G  R  W  L  V  I  A  L  I  Q  L  A  K
 346. ATGGGAGCCGCCAAAGTGTGGGGCGAAGTGGGACGCTGGCTCGTGATCGCCCTGATCCAGCTGGCCAAG
      A  V  L  R  M  L  L  L  L  W  F  K  A  G  L  Q  T  S  P  P  I  V  P
 415. GCCGTGCTGCGCATGTTGCTGCTGCTGTGGTTCAAGGCCGGACTGCAGACCAGCCCCCCAATCGTGCCA
      L  D  R  E  T  Q  A  Q  P  P  D  G  D  H  S  P  G  N  H  E  Q  S  Y
 484. CTGGATCGCGAGACGCAGGCCCAGCCACCAGATGGCGATCACTCCCCAGGCAACCACGAGCAGAGCTAC
      V  G  K  R  S  N  R  V  V  R  T  L  Q  N  T  P  S  L  H  S  R  H  W
 553. GTGGGCAAGCGCTCCAATCGCGTCGTGCGCACCCTGCAGAATACCCCCAGTCTGCACAGCCGCCATTGG
      G  A  P  Q  Q  R  E  G  R  Q  Q  Q  H  H  E  E  L  S  A  T  P  T  P
 622. GGAGCCCCCCAGCAGCGCGAGGGACGCCAGCAGCAGCATCACGAGGAGCTGAGTGCCACCCCAACCCCA
      L  G  L  Q  E  T  I  A  E  F  L  Y  I  A  R  P  L  L  H  L  L  S  L
 691. CTGGGACTGCAGGAGACGATCGCCGAGTTCCTGTACATTGCCCGCCCACTGCTGCATCTGCTGAGCCTG
      G  L  W  G  Q  R  S  W  K  P  W  L  L  A  G  V  V  D  V  T  S  L  S
 760. GGACTGTGGGGACAGCGCAGTTGGAAGCCCTGGCTGCTGGCCGGCGTGGTGGATGTGACCAGTCTGAGC
      L  L  S  D  R  K  G  L  T  R  R  E  R  R  E  L  R  R  R  T  I  L  L
 829. CTGCTGAGCGATCGCAAGGGACTGACCCGCCGCGAGCGCCGCGAGCTGCGCCGCCGCACCATTCTGCTG
      L  Y  Y  L  L  R  S  P  F  Y  D  R  F  S  E  A  R  I  L  F  L  L  Q
 898. CTGTACTATCTGCTGCGCTCCCCCTTCTACGATCGCTTCTCCGAGGCCCGCATCCTGTTTCTGCTGCAG
      L  L  A  D  H  V  P  G  V  G  L  V  T  R  P  L  M  D  Y  L  P  T  W
 967. CTGCTGGCCGATCACGTGCCCGGCGTGGGACTCGTGACGCGCCCACTGATGGATTACCTGCCCACCTGG
      Q  K  I  Y  Y  S  W  G  *
1036. CAGAAGATCTACTACAGCTGGGGCTAACTCGAG
```

Disease Models & Mechanisms • Supplementary information

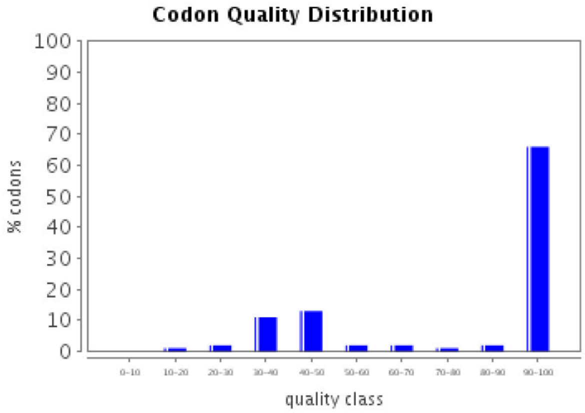
Codon Quality Distribution
% codons
quality class

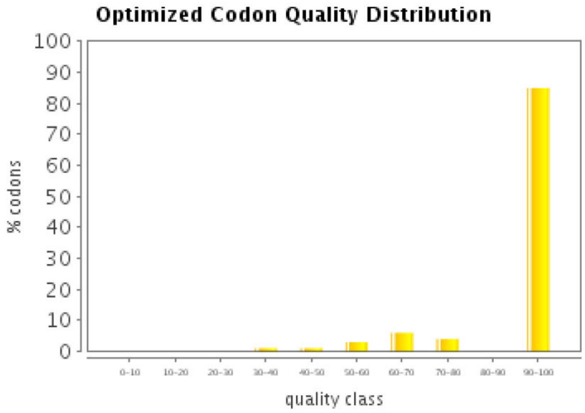
Optimized Codon Quality Distribution
% codons
quality class

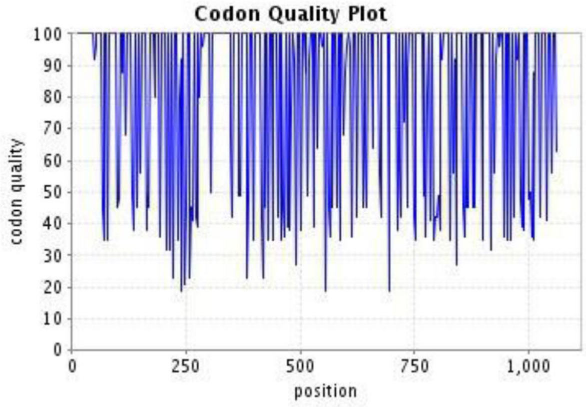
Codon Quality Plot
codon quality
position

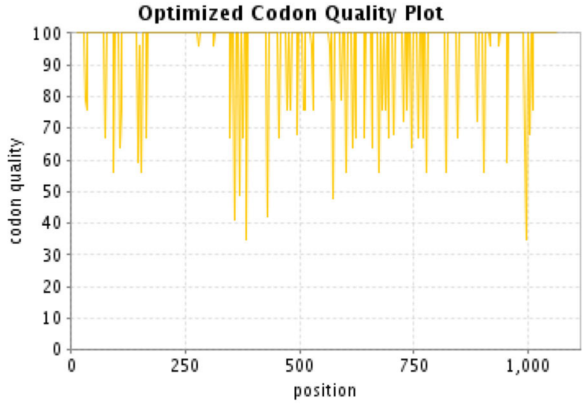
Optimized Codon Quality Plot
codon quality
position

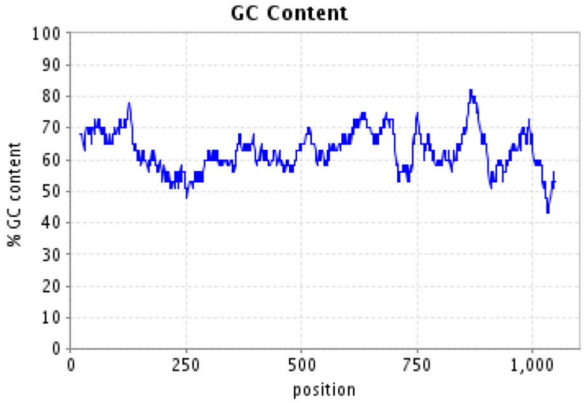
GC Content
% GC content
position

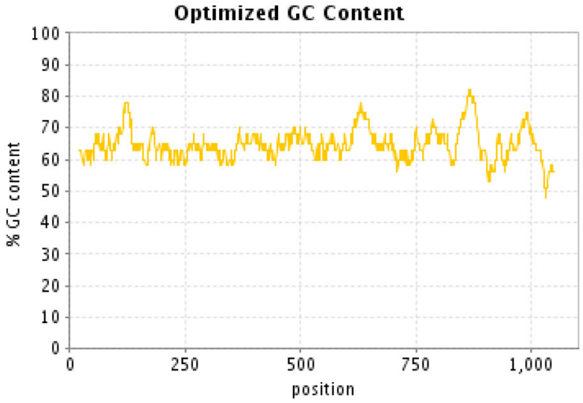
Optimized GC Content
% GC content
position

```
    MASRKENAKSANRVLRISQLDALELNKALEQLVWSQFTQCFHGFKPGLLARFEPEV  56
KACLWVFLWRFTIYSKNATVGQSVLNIKYKNDFSPNLRYQPPSKNQKIWYAVCTIGGRWL  116
EERCYDLFRNHHLASFGKVKQCVNFVIGLLKLGGLINFLIFLQRGKFATLTERLLGIHSV  176
FCKPQNIREVGFEYMNRELLWHGFAEFLIFLLPLINVQKLKAKLSSWCIPLTGAPNSDNT  236
LATSGKECALCGEWPTMPHTIGCEHIFCYFCAKSSFLFDVYFTCPKCGTEVHSLQPLKSG  296
IEMSEVNALVSKGEELFTGVVPILVELDGDVNGHKFSVSGEGEGDATYGKLTLKFICTTG  356
KLPVPWPTLVTTLTYGVQCFSRYPDHMKQHDFFKSAMPEGYVQERTIFFKDDGNYKTRAE  416
VKFEGDTLVNRIELKGIDFKEDGNILGHKLEYNYNSHNVYIMADKQKNGIKVNFKIRHNI  476
EDGSVQLADHYQQNTPIGDGPVLLPDNHYLSTQSALSKDPNEKRDHMVLLEFVTAAGITL  536
GMDELYK  543
```

PEX2_E55K

```
    MASRKENAKSANRVLRISQLDALELNKALEQLVWSQFTQCFHGFKPGLLARFEPKV  56
KACLWVFLWRFTIYSKNATVGQSVLNIKYKNDFSPNLRYQPPSKNQKIWYAVCTIGGRWL  116
EERCYDLFRNHHLASFGKVKQCVNFVIGLLKLGGLINFLIFLQRGKFATLTERLLGIHSV  176
FCKPQNIREVGFEYMNRELLWHGFAEFLIFLLPLINVQKLKAKLSSWCIPLTGAPNSDNT  236
LATSGKECALCGEWPTMPHTIGCEHIFCYFCAKSSFLFDVYFTCPKCGTEVHSLQPLKSG  296
IEMSEVNALVSKGEELFTGVVPILVELDGDVNGHKFSVSGEGEGDATYGKLTLKFICTTG  356
KLPVPWPTLVTTLTYGVQCFSRYPDHMKQHDFFKSAMPEGYVQERTIFFKDDGNYKTRAE  416
VKFEGDTLVNRIELKGIDFKEDGNILGHKLEYNYNSHNVYIMADKQKNGIKVNFKIRHNI  476
EDGSVQLADHYQQNTPIGDGPVLLPDNHYLSTQSALSKDPNEKRDHMVLLEFVTAAGITL  536
GMDELYK  543
```

PEX2_C247R

```
    MASRKENAKSANRVLRISQLDALELNKALEQLVWSQFTQCFHGFKPGLLARFEPEV  56
KACLWVFLWRFTIYSKNATVGQSVLNIKYKNDFSPNLRYQPPSKNQKIWYAVCTIGGRWL  116
EERCYDLFRNHHLASFGKVKQCVNFVIGLLKLGGLINFLIFLQRGKFATLTERLLGIHSV  176
FCKPQNIREVGFEYMNRELLWHGFAEFLIFLLPLINVQKLKAKLSSWCIPLTGAPNSDNT  236
LATSGKECALRGEWPTMPHTIGCEHIFCYFCAKSSFLFDVYFTCPKCGTEVHSLQPLKSG  296
IEMSEVNALVSKGEELFTGVVPILVELDGDVNGHKFSVSGEGEGDATYGKLTLKFICTTG  356
KLPVPWPTLVTTLTYGVQCFSRYPDHMKQHDFFKSAMPEGYVQERTIFFKDDGNYKTRAE  416
VKFEGDTLVNRIELKGIDFKEDGNILGHKLEYNYNSHNVYIMADKQKNGIKVNFKIRHNI  476
EDGSVQLADHYQQNTPIGDGPVLLPDNHYLSTQSALSKDPNEKRDHMVLLEFVTAAGITL  536
GMDELYK  543
```

PEX2_W223X

```
    MASRKENAKSANRVLRISQLDALELNKALEQLVWSQFTQCFHGFKPGLLARFEPEV  56
KACLWVFLWRFTIYSKNATVGQSVLNIKYKNDFSPNLRYQPPSKNQKIWYAVCTIGGRWL  116
EERCYDLFRNHHLASFGKVKQCVNFVIGLLKLGGLINFLIFLQRGKFATLTERLLGIHSV  176
FCKPQNIREVGFEYMNRELLWHGFAEFLIFLLPLINVQKLKAKLSSVSKGEELFTGVVPI  236
LVELDGDVNGHKFSVSGEGEGDATYGKLTLKFICTTGKLPVPWPTLVTTLTYGVQCFSRY  296
PDHMKQHDFFKSAMPEGYVQERTIFFKDDGNYKTRAEVKFEGDTLVNRIELKGIDFKEDG  356
NILGHKLEYNYNSHNVYIMADKQKNGIKVNFKIRHNIEDGSVQLADHYQQNTPIGDGPVL  416
LPDNHYLSTQSALSKDPNEKRDHMVLLEFVTAAGITLGMDELYK  460
```

Disease Models & Mechanisms • Supplementary information

PEX2_R119X

```
    MASRKENAKSANRVLRISQLDALELNKALEQLVWSQFTQCFHGFKPGLLARFEPEV  56
KACLWVFLWRFTIYSKNATVGQSVLNIKYKNDFSPNLRYQPPSKNQKIWYAVCTIGGRWL  116
EEVSKGEELFTGVVPILVELDGDVNGHKFSVSGEGEGDATYGKLTLKFICTTGKLPVPWP  176
TLVTTLTYGVQCFSRYPDHMKQHDFFKSAMPEGYVQERTIFFKDDGNYKTRAEVKFEGDT  236
LVNRIELKGIDFKEDGNILGHKLEYNYNSHNVYIMADKQKNGIKVNFKIRHNIEDGSVQL  296
ADHYQQNTPIGDGPVLLPDNHYLSTQSALSKDPNEKRDHMVLLEFVTAAGITLGMDELYK  356
```

```
    MGKPIPNPLLGLDSTEKLRLLGLRYQEYVTRHPAATAQLETAVRGFSYLLAGRFAD  56
SHELSELVYSASNLLVLLNDGILRKELRKKLPVSLSQQKLLTWLSVLECVEVFMEMGAAK  116
VWGEVGRWLVIALIQLAKAVLRMLLLLWFKAGLQTSPPIVPLDRETQAQPPDGDHSPGNH  176
EQSYVGKRSNRVVRTLQNTPSLHSRHWGAPQQREGRQQQHHEELSATPTPLGLQETIAEF  236
LYIARPLLHLLSLGLWGQRSWKPWLLAGVVDVTSLSLLSDRKGLTRRERRELRRRTILLL  296
YYLLRSPFYDRFSEARILFLLQLLADHVPGVGLVTRPLMDYLPTWQKIYFYSWG  350
```

PEX16_R176X

```
    MGKPIPNPLLGLDSTEKLRLLGLRYQEYVTRHPAATAQLETAVRGFSYLLAGRFAD  56
SHELSELVYSASNLLVLLNDGILRKELRKKLPVSLSQQKLLTWLSVLECVEVFMEMGAAK  116
VWGEVGRWLVIALIQLAKAVLRMLLLLWFKAGLQTSPPIVPLDRETQAQPPDGDHSPGNH  176
EQSYVGKRSNRVV*  193
```

PEX16_del_955TCT

```
    MGKPIPNPLLGLDSTEKLRLLGLRYQEYVTRHPAATAQLETAVRGFSYLLAGRFAD  56
SHELSELVYSASNLLVLLNDGILRKELRKKLPVSLSQQKLLTWLSVLECVEVFMEMGAAK  116
VWGEVGRWLVIALIQLAKAVLRMLLLLWFKAGLQTSPPIVPLDRETQAQPPDGDHSPGNH  176
EQSYVGKRSNRVVRTLQNTPSLHSRHWGAPQQREGRQQQHHEELSATPTPLGLQETIAEF  236
LYIARPLLHLLSLGLWGQRSWKPWLLAGVVDVTSLSLLSDRKGLTRRERRELRRRTILLL  296
YYLLRSPFYDRFSEARILFLLQLLADHVPGVGLVTRPLMDYLPTWQKIY_YSWG  349
```

Fig. S4. Generation of human UAS-*PEX2* and UAS-*PEX16* lines for reference and variant constructs with the human protein encoded from GeneART (Thermo Fisher Scientific).

Disease Models & Mechanisms • Supplementary information

Table S1. *Drosophila Pex2* & *Pex16* survival curve comparison test.
(**A**) Indicates the results of the Log-rank test survival curve comparison tests of both female and male *Pex2* lifespan. (**B**) Indicates the results of the Log-rank test survival curve comparison tests of both female and male *Pex16* lifespan. [* = *p*-value is less than 0.05. ** = *p*-value is less than 0.01. *** = *p*-value is less than 0.001. **** = *p*-value is less than 0.0001]

Available for download at
https://journals.biologists.com/dmm/article-lookup/doi/10.1242/dmm.052258#supplementary-data

Table S2. Human *PEX2* & *PEX16* survival curve comparison test.
(**A**) Indicates the results of the Log-rank test tests of the *PEX2* (male and female), female only *PEX2,* and male only *PEX2* survival curves between all genotypes. (**B**) Indicates the results of the Log-rank test tests of the *PEX16* (male and female), female only *PEX16,* and male only *PEX16* survival curves between all genotypes. [* = *p*-value is less than 0.05. ** = *p*-value is less than 0.01. *** = *p*-value is less than 0.001. **** = *p*-value is less than 0.0001]

Available for download at
https://journals.biologists.com/dmm/article-lookup/doi/10.1242/dmm.052258#supplementary-data

Table S3. Observed/expected Mendelian ratio calculation of the F1 generation of human *PEX2* and *PEX16* crosses.

Available for download at
https://journals.biologists.com/dmm/article-lookup/doi/10.1242/dmm.052258#supplementary-data
